# A diastereoselective synthesis of Cebranopadol, a novel analgesic showing NOP/mu mixed agonism

**DOI:** 10.1038/s41598-017-02502-9

**Published:** 2017-05-25

**Authors:** Anna Fantinati, Sara Bianco, Remo Guerrini, Severo Salvadori, Salvatore Pacifico, Maria Camilla Cerlesi, Girolamo Calo, Claudio Trapella

**Affiliations:** 1 Department of Chemical and Pharmaceutical Sciences and LTTA, University of Ferrara, 44121 Ferrara, Italy; 2 Department of Medical Sciences, Section of Pharmacology and National Institute of Neuroscience, University of Ferrara, 44121 Ferrara, Italy

## Abstract

A diastereoselective synthesis of the title compound as a single *E* diastereomer has been efficiently accomplished by assembling the featured pyrano-indole scaffold of the spiro[cyclohexane-dihydropyrano[3,4-b]-indole]-amine framework through an oxa-Pictet-Spengler reaction, promoted by a cheap and green Zeolite catalyst. Basic pharmacological experiments demonstrate that Cebranopadol acts as a mixed nociception/orphanin FQ (NOP) and mu (MOP) opioid receptor agonist useful for treatment of chronic pain.

## Introduction

**Figure 1:**
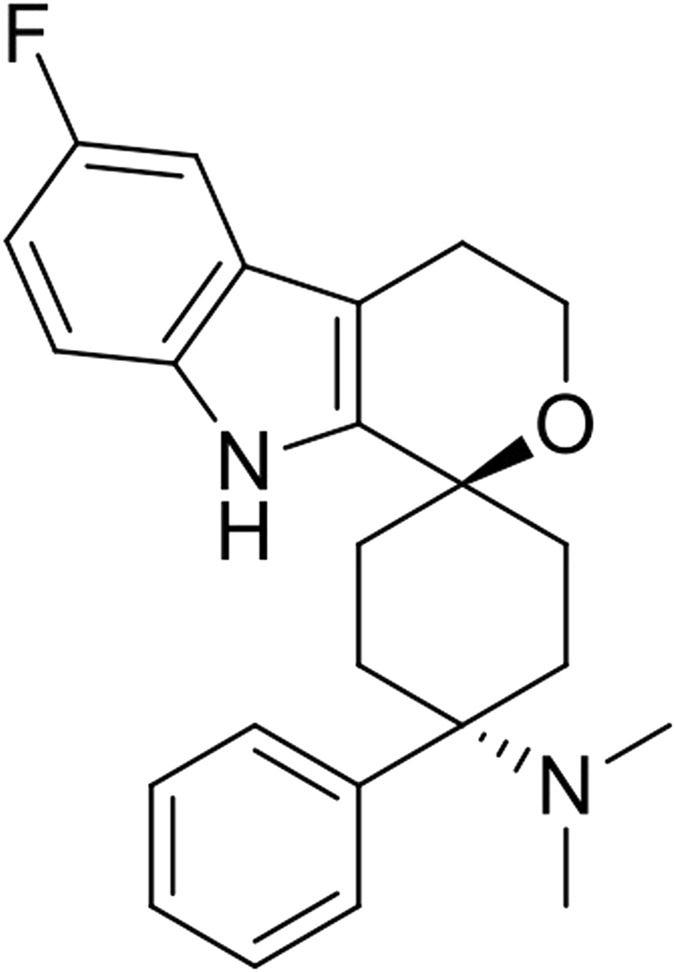
Cebranopadol.

Nociceptin/orphanin FQ (N/OFQ), the endogenous agonist of the N/OFQ peptide receptor (NOP) regulates various biological functions^1^ including pain transmission^2^. Grünenthal researchers have recently reported the results of structure activity studies^3, 4^ that led to the identification of Cebranopadol (trans-6′-fluoro-4′-9′-dihydro-N,N-dimethyl-4-phenyl-spiro[ciclohexane-1,1′(3′H)-pyrano[3,4-b]indol]-4-amine) as a potent NOP and mu receptor (MOP) agonist (Fig. 1).

This compound binds with high affinity to the NOP and the MOP receptors and behaves as full agonist. Rodent studies demonstrated that Cebranopadol elicits potent and efficacious antinociceptive action in several models of nociceptive and neuropathic pain. Importantly, compared to morphine, Cebranopadol displayed a favorable side effect profile and reduced tolerance liability^5^. Cebranopadol is under clinical development and several clinical trials are assessing its analgesic therapeutic potential in different pain conditions^6^.

## Results and Discussion

**Figure 2:**
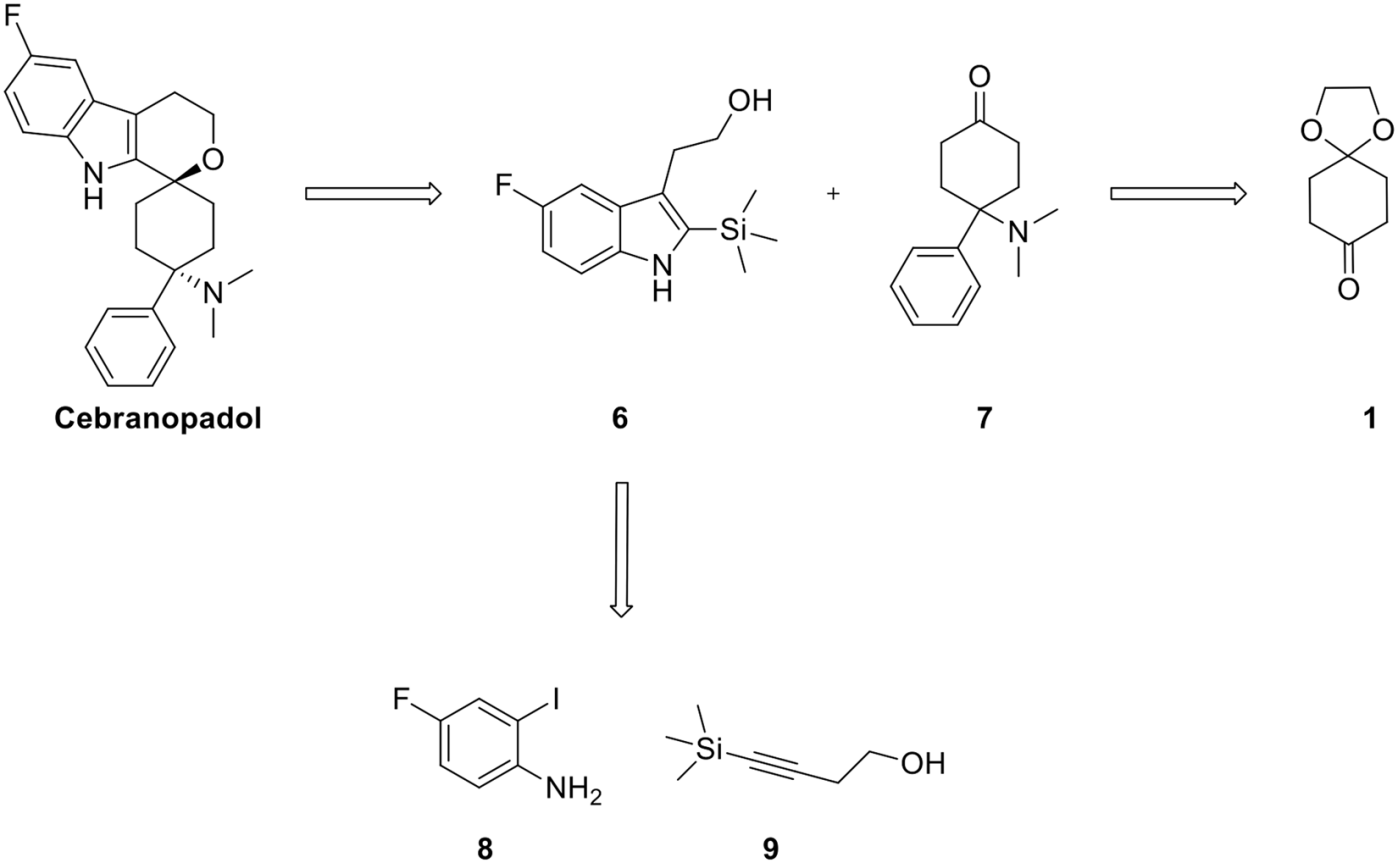
Retrosynthesis of Cebranopadol.

Grünenthal researchers synthetized Cebranopadol as retrosynthetically depicted in Fig. 2 based on a oxa-PictetSpengler reaction between the fluoro-silyl-indole **6**, in turn prepared by Larock indole synthesis^7^ of the commercially available 2-iodo-4-fluoroaniline **8** and 1-silyl-1-butynol **9**, and the aminoketone **7** bearing the structurally important 4-N,Ndimethylamino-4-phenyl-cyclohexane head by a two step approach involving a Strecker synthesis^8^ of monoketal protected 1,4-cyclohexanedione **1** by treatment with HNMe_2_. HCl/KCN (67–99%) and a Bruylants reaction^9^ of the resulting aminonitrile with PhMgCl in THF at 0 °C to room temperature (low overall yield). It deals with a classical approach that at the end of the synthesis requires a diastereomer separation by HPLC.

**Figure 3:**
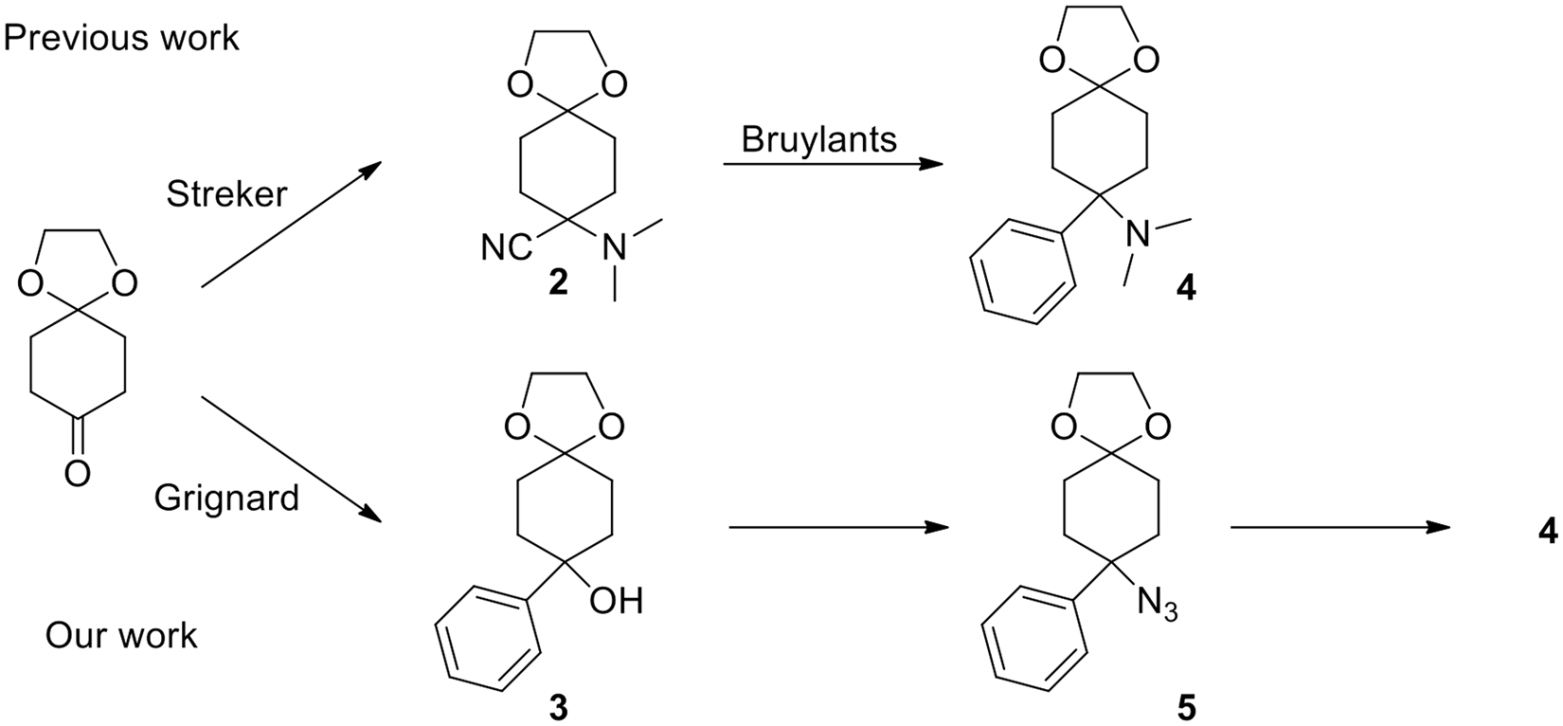
Different approaches to the synthesis of Cebranopadol.

Our interest in this area led us to be contemporaneously involved in the synthesis of Cebranopadol with two important guidelines in our project developed along the same pathway: a) avoid the use of highly toxic potassium cyanide; b) identifying suitable experimental conditions to make the oxa-PictetSpengler reaction diastereoselective. (Fig. 3).

**Figure 4:**
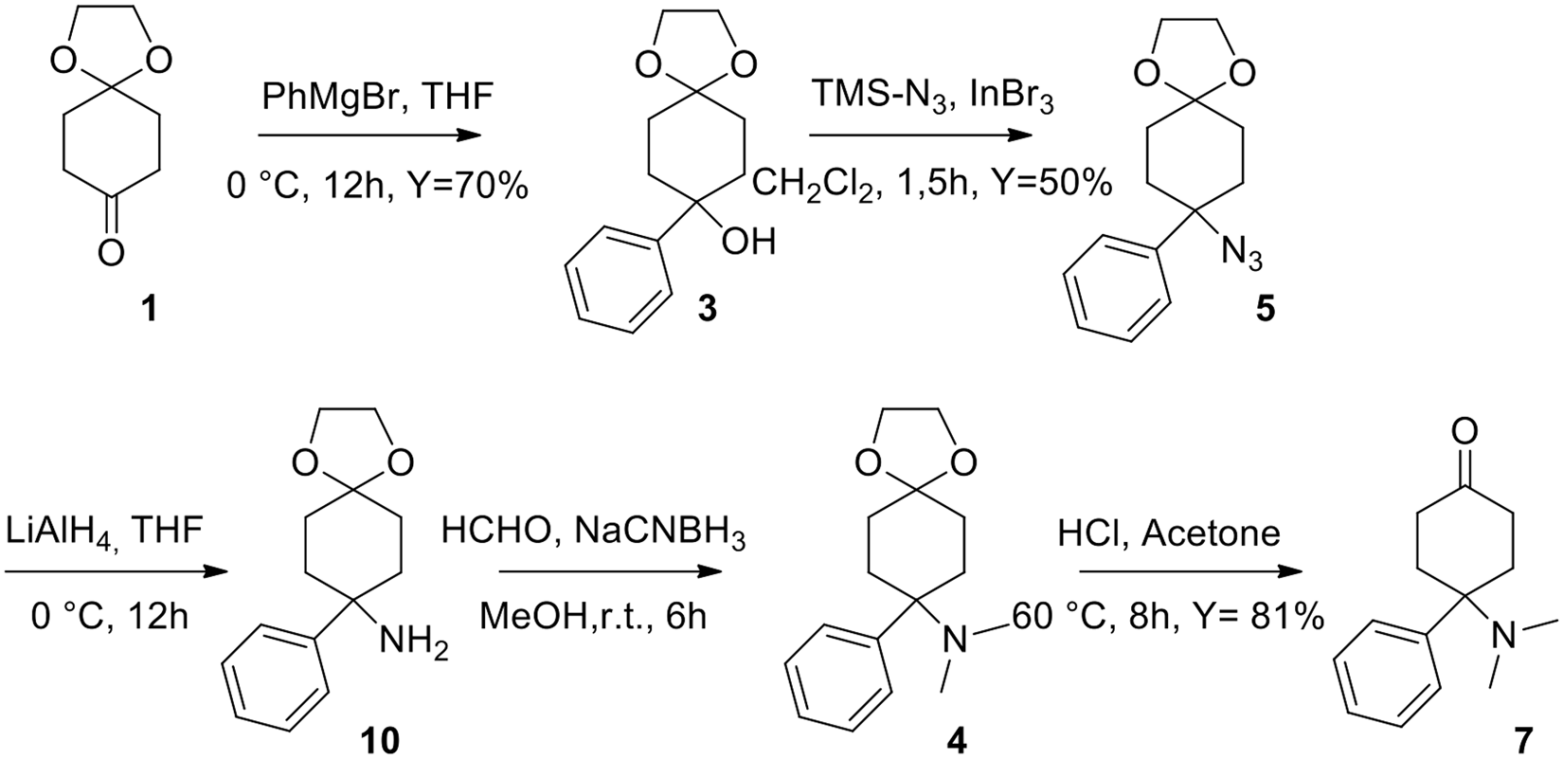
Synthesis of intermediate 7.

The approach proposed a start from the cheap and commercially available ketone **1** that undergoes a Grignard reaction with phenyl magnesium bromide in THF for 12 h yielding the corresponding alcohol **3** at 70% yield. A nucleophilic substitution of the tertiary and benzylic alcohol using the classical sodium azide/TFA in chloroform approach failed. Different methods have been tried to overcome the very low yield (5%) because of the competitive elimination reaction to an alkene. In our hands the best way to obtain the azide **5** was the reaction with trimethylsilylazide catalysed by indium tribromide^10^ that allowed us to obtain compound **5** in a clean and fast step with good yield (50%). Lithium aluminium hydride reduction to the primary amine **10** followed by reductive amination with formaldehyde allowed us to obtain the tertiary amine **4** in good overall yield. Deprotection of the ketone using hydrochloric acid in acetone produced the intermediate **7** (Fig. 4). The stage was set for the crucial oxa-Pictet-Spengler reaction which has been performed in different conditions such as bismuth triflate^11, 12^, hydrochloric acid, Zeolite beta-25 and 4 Å molecular sieves giving rise to a mixture of diastereomers in low yield. The cyclization reaction has been tried also using a TMSOTf at room temperature producing Cebranopadol in 90% yield but lacking stereoselectivity^4^.

**Figure 5:**
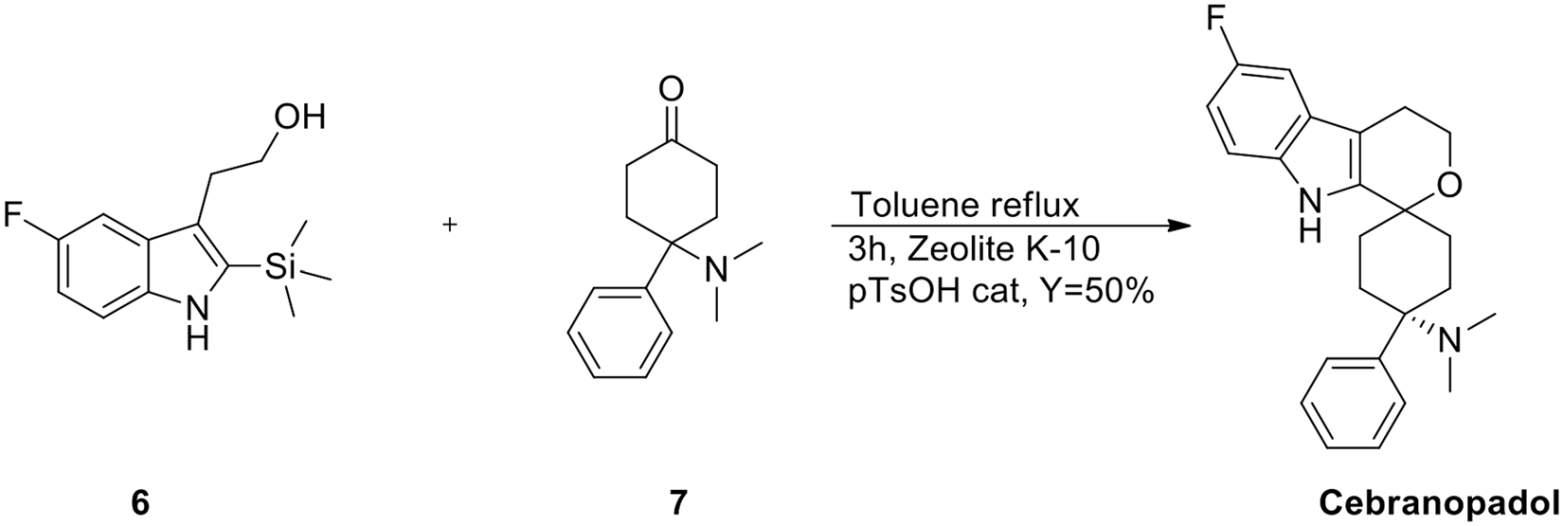
Unusual diastereoselective oxa-Pictet-Spengler reaction.

Much to our delight, the last synthetic steps involving an oxaPictet-Spengler reaction to install the dihydropyrano[3,4-b]indole moiety have been performed in a diastereoselective manner using an unusual and green Zeolite catalyst^11^ and catalytic *p*-toluenesulphonic acid in refluxing toluene that allowed us to obtain the E diastereomer exclusively as depicted in Fig. 5.

The setting up of an oxa-Pictet-Spengler reaction, allowed us to also obtain different pyrano indole scaffolds starting from commercially and non commercially available ketones. ROESY NMR experiments (see Supporting Information page﻿ S20)of the final compound confirms that the zeolite K-10 catalyst produced a single E diastereomer during the oxa-Pictet-Spengler reaction. On the contrary, the trimethylsilyl triflate approach allowed us to obtain a mixture of E and Z diastereomer with a good overall yield but without regioselectivity. The zeolite catalyst is easily recovered and its catalytic properties did not change up to 5 times reusing-cycle.

**Figure 6:**
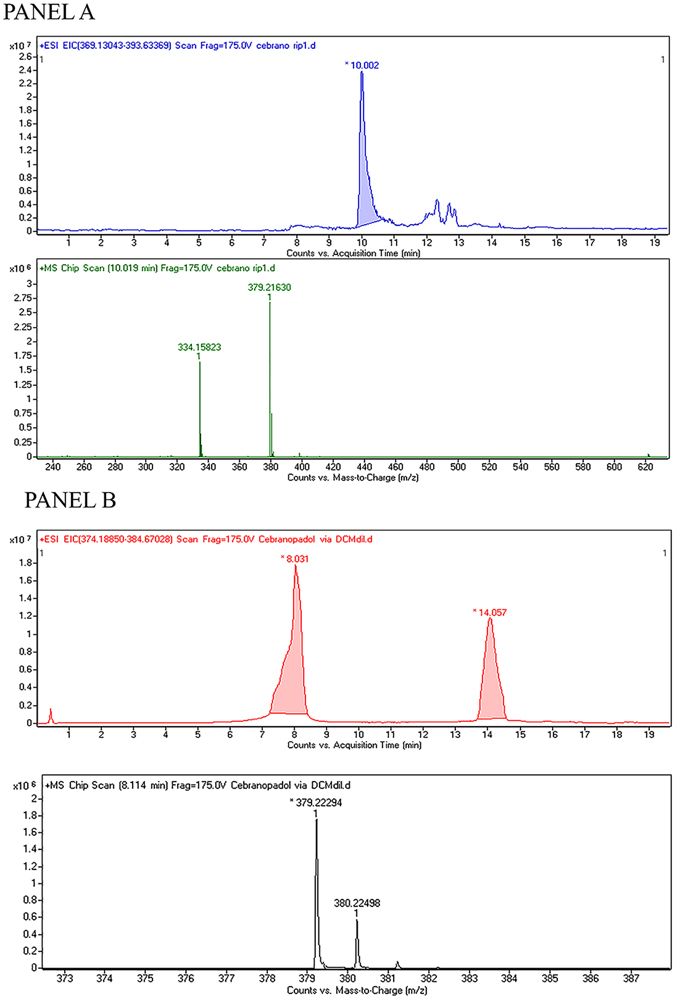
The HR-LC-MS spectra of Cebranopadol.

HR-LC-MS spectra of Cebranopadol obtained using Zeolite catalyst (panel A) and TMSOTf (panel B) are depicted in Fig. 6.

The basic pharmacological profile of Cebranopadol has been investigated by measuring calcium mobilization in cells coexpressing NOP or classical opioid receptors and chimeric G proteins as described in detail in Camarda *et* *al*.^13, 14^.

The results of these experiments, summarized in Table 1, indicated that Cebranopadol behaved as full agonist showing very similar potency at NOP and MOP receptors. Cebranopadol was also able to activate kappa (KOP) and delta (DOP) opioid receptors but with lower potency and, in the case of the KOP receptor, lower efficacy. These findings are in line with those reported by Grünenthal researchers in receptor binding and stimulated [^35^S]GTPgS binding experiments^5^.

**Table 1:** Pharmacological profile of Cebranopadol (Cebra) in cells coexpressing human recombinant NOP or classical opioid receptors and chimeric G protein.

	NOP		MOP		KOP		DOP	
	pEC_50_	α	pEC_50_	α	pEC_50_	α	pEC_50_	α
**N/OFQ**	9.59	1.00	inactive		inactive		inactive	
**Fentanyl**	inactive		8.13	1.00	inactive		inactive	
**Dyn A**	inactive		6.67	0.82	8.54	1.00	7.73	0.99
**DPDPE**	inactive		inactive		inactive		8.15	1.00
**Cebra**	7.28	0.89	7.20	0.99	5.98	0.55	6.31	0.81

## Conclusions

In conclusion, we have developed a robust and easy method for the synthesis of Cebranopadol (in 15% overall yield) as a single E diastereomer using a green Zeolite catalyst in the key oxaPictet-Spengler reaction. The azide approach will allow us to easily insert different substituents onto the basic amine to better understand the crucial role of tertiary amines in the interaction with Asp^130^ and Asp^147^ of the NOP and MOP receptor binding pockets. Basic pharmacological experiments demonstrate that Cebranopadol acts as a mixed NOP/MOP agonist.

## Methods

### Chemical Materials and Methods

All NMR spectra were analysed using Mestre Nova 6.0.2 software and FID data are available on request. Analytical thin layer chromatography (TLC) was performed on silica gel Macherey-Nagel poligram SIL/UV 254 of 0.25 mm, visualization was achieved using UV light (254) and Potassium Permanganate (KMnO_4_) 2% in water. Flash column chromatography was undertaken using Isolera one (Biotage Sweden). Products were dried using sodium sulfate anhydrous Carlo Erba. Proton nuclear magnetic resonance (^1^H NMR) and carbon nuclear magnetic resonance (^13^C NMR) were recorded using VARIAN 400 MHz. All spectra were recorded using CDCl_3_ as a solvent otherwise the solvent was specified. Chemical shifts (δ) were quoted in ppm relative to residual solvent and coupling constants (J) were quoted in Hertz (Hz). Multiplicity was reported with the following abbreviations: s = singlet; d = doublet; t = triplet; q = quartet; m = multiplet; dd = doublet of doublet; dt = doublet of triplet, dq = doublet of quartet; Infrared (IR) spectra were recorded on a Perkin-Elmer FT-IR Spectrum 100 using zirconium-selenium diamond as a cell. Melting points were recorded using a Buchi-Tottoli and were reported uncorrected. Molecular weights were measured with a mass spectrometer electrospray ESI MICROMASS ZMD 2000 and high resolution spectra with an Agilent ESI-Q-TOF LC/MS 6520 System. Solvents and chemicals used for TLC, chromatographic purification, crystallization and reactions were reported with the following abbreviations: Et_2_O for diethyl ether, THF for tetrahydrofuran, AcOEt for ethyl acetate, DCM for methylene chloride, LiAlH_4_ for lithium aluminium hydride.

### Synthesis of 8-phenyl-1,4-dioxaspiro[4.5]decan-8-ol (3)

In a two neck round bottom flask, under an argon atmosphere, 1,4-cyclohexanedione monoethylene acetale (**1**) (1.5 g, 9.6 mmol) was dissolved in THF (50 mL). At 0 °C, phenyl magnesium bromide (20 ml, 19.22 mmol) was added and the reaction was stirred overnight at room temperature. The reaction mixture was checked by TLC (AcOEt/Petroleum ether 1:6), quenched with NH_4_Cl saturated solution and washed with AcOEt. The organic layers were dried with Na_2_SO_4_, filtered and the solvent was removed under vacuum. The crude product was purified by flash chromatography (AcOEt/ Petroleum ether 1:1) with an yield of 70% to give the title compound (**3**) as a white solid. MS (ESI): [M-OH]^+^. = 217.27, m. p.: 98–100 °C

^1^H-NMR (400 MHz, Chloroform-d), δ: 7.58–7.46 (m, 1 H, Ar), 7.41–7.31 (m, 2 H, Ar), 7.30–7.22 (m, 2 H, Ar), 4.03–3.91 (m, 4 H, O-CH_2_-CH_2_-O-), 2.24–2.05 (m, 4 H, CH_2_ cyclohexane), 1.86–1.77 (m, 2 H CH_2_ cyclohexane), 1.74–1.64 (m, 3 H CH_2_ cyclohexane and -OH). ^13^C-NMR (100 MHz, Chloroform-d), δ: 148.59 (Cq-Ar), 128.74, 128.42, 127.06, 124.64, (CH-Ar), 108,55 (-O-Cq-O), 72.57 (Ar-Cq-OH), 64.49 (-O-CH_2_-CH_2_-O), 64.38 (-O-CH_2_-CH_2_-O), 36.73 (CH_2_ cyclohexane), 30.89 (CH_2_ cyclohexane).

### Synthesis of 8-azido-8-phenyl-1,4-dioxaspiro[4.5]decane (5)

In a round bottom flask, compound (**3**) (1 g, 4.27 mmol) was disolved in DCM (30 mL) and then trimehylsilyl azide (1.13 ml, 6.41 mmol) and indium tribromide (151.38 mg, 0.427 mmol) were added. The reaction mixture was stirred for 1 hour and then worked up with NaHCO_3_ until basic in pH. The reaction mixture was extracted twice with 30 mL of DCM each time, the organic layers were dried, filtered and concentrated. The crude product was purified by flash chromatography on silica gel in AcOEt/petroleum ether 1:3 to give the title compound (**5**) with a yield of 50% as a pale yellow oil. MS (ESI): [M-N_3_]^+^  = 217.25; 1H-NMR (400 MHz, Chloroform-d), δ: 7.57–7.11 (m, 5 H, Ar), 4.02–3.93 (m, 4 H, O-CH_2_-CH_2_-O), 2.28–1.82 (m, 6 H, CH_2_ cyclohexane), 1.88–1.49 (m, 2 H, CH_2_ cyclohexane). ^13^C-NMR (100 MHz, Chloroform-d), δ: 143.36 (Cq-Ar), 128.81, 128.81, 127.63, 125.54 (CH-Ar), 107.89 (-O-Cq-O-), 65.90 (Cq-N_3_), 64.59 (-O-CH_2_-CH_2_-O), 64.41 (-O-CH_2_-CH_2_-O), 33.74 (CH_2_ cyclohexane), 31.19 (CH_2_ cyclohexane).

### Synthesis of 8-phenyl-1,4-dioxaspiro[4.5]decan-8-amine (10)

To a solution of LiAlH_4_ (437 mg, 11.53 mmol) in 15 mL of THF, was added the azide (**5**) (1 g, 3.83 mmol) disolved in the same solvent at 0 °C. The reaction mixture was stirred overnight and was monitored by ESI-mass and TLC (AcOEt/Petroleum ether 1:3), worked up with NaOH 5%(10 mL) and filtered over celite pad in Et_2_O. The solvent was removed under vacuum to give the compound (**10**) in quantitative yield pure enough to be used without purification in the next step. MS (ESI): [M + H]^+^ = 234.36, [M-NH_2_]^+^. = 217.39.

### Synthesis of N,N-dimethyl-8-phenyl-1,4-dioxaspiro[4.5]decan-8-amine (4)

To a solution of compound (**10**) (1 g, 3.71 mmol) in 50 mL of MeOH, formaldehyde (1.04 ml, 37.17 mmol), sodium triacetoxyborohydride (1.57 g, 7.43 mmol) and a catalytic amount of AcOH were added at room temperature. The reaction mixture was stirred overnight at room temperature until the formation of the title compound. The solvent was concentrated to dryness, diluted in AcOEt and the organic layers were washed with NaOH 5% solution (2 × 20 mL) in order to obtain the tertiary amine (**4**) as a colourless sticky solid with a quantitative yield. The tertiary amine was purified by flash chromatography (eluent AcOEt/EtPt 3:1). MS (ESI): [M + H]^+^ = 262.35; HRMS (ESI): [M + H]^+^ Calc. = 262.180155; [M + H]^+^ Found = 262.18122. ^1^H NMR (400 MHz, Chloroform-d), δ: 7.34 (m, 5 H, Ar), 3.95 (ddd, 2 H, J = 6.1, 5.6, 1.4, -O-CH_2_-CH_2_-O), 3.89 (m, 2 H, -O-CH_2_-CH_2_-O), 2.31 (m, 2 H, CH_2_ Cyclohexyl), 2.16 (m, 2 H, CH_2_ Cyclohexyl), 2.09 (s, 6 H, N(CH_3_)_2_), 1.80 (m, 2 H, CH_2_ Cyclohexyl), 1.49, (ddd, 2 H, J = 13.9, 10.9, 3.7, CH_2_ Cyclohexyl). ^13^C-NMR (100 MHz, Chloroform-d), δ:128.30, 128.00, 127.88, 127.63, 125.33 (C-Ar), 108.75 (O-Cq-O) 64.62 (O-CH_2_-CH_2_-O), 64.38 (O-CH_2_-CH_2_-O), 48.82 (Cq-N(CH_3_)_2_), 38.44 (N(CH_3_)_2_), 31.37 (CH_2_ Cyclohexyl), 30.38 (CH_2_ Cyclohexyl).

### Synthesis of 4-(dimethylamino)-4-phenylcyclohexan-1-one (7)

To a stirred solution of tertiary amine (**4**) (1 g, 3.81 mmol) in 70 mL of acetone, hydrochloric acid 10% was added to achieve an acid pH, the reaction mixture was stirred overnight at 65 °C. The reaction mixture was monitored by ESI-mass spectrometry (peak 218) and TLC (AcOEt/Petroleum ether /NH3 3:1: 0.3). The solvent was removed under vacuum and the residue diluted in 50 mL of AcOEt. The organic layers were washed with NaOH 10% (2 × 20 mL), dried, filtered and concentrated to give a crude product purified by flash chromatography on silica gel using as solvents AcOEt/Petroleum ether/NH3 3:1:0.3 to give the title compound (**7**) as a white solid with a yield of 81%. HRMS (ESI): [M + H]^+^ Calc. = 218.153941; [M + H]^+^ Found = 218.15313. ^1^H NMR (400 MHz, Chloroform-d), δ: 7.43 (m, 5 H, CH-Ar), 2.65–2.57 (m, 4 H, CH_2_ Cyclohexyl), 2.37–2.24 (m, 4 H, CH_2_ Cyclohexyl), 2.19 (s, 6 H, N(CH_3_)_2_). ^13^C NMR (100 MHz, Chloroform-d), δ: 210.92 (C=O), 128.40, 127.74, 127.57, 125.33 (C-Ar), 62.92 (Cq-N(CH_3_)_2_), 38.43 (N(CH_3_)_2_), 37.34 (CH_2_ Cyclohexyl), 32.54 (CH_2_ Cyclohexyl).

### Synthesis of 2-(5-fluoro-2-(trimethylsilyl)-1H-indol-3-yl)ethan-1-ol (6)

In a two neck round bottom flask, under an argon atmosphere, the 4-fluoro-2-iodoaniline (**8**) (2 g, 8.43 mmol) was disolved in 15 mL of DMF. 4-(trimethylsilyl)but-3-yn-1-ol (**9**) (1.55 ml, 9.27 mmol), potassium carbonate (1.165 g, 8.43 mmol), lithium chloride (357 mg, 8.43 mmol), triphenylphosphine (144 mg, 0.42 mmol), palladium acetate (94 mg, 0.42 mmol) were added and the reaction was stirred overnight under reflux. The reaction was monitored by TLC (AcOEt/Petroleum ether 1:4) and the solvent was removed under vacuum. The residue was dissolved in 30 mL of AcOEt and washed twice with brine (10 mL each), the organic layers were dried, filtered and concentrated in vacuo. The crude product was purified by flash chromatography with AcOEt/Petroleum ether 1:4 with a yield of 40% to give the title compound (**6**) as a yellow liquid. MS (ESI): [M + H]^+^ = 252.22; ^1^H-NMR (400 MHz, Chloroform-d), δ: 8.21–8.14 (bs, 1 H, NH), 7.28–7.22 (m, 1 H, CH-Ar), 7.09–7.05 (m, 1 H, CH-Ar), 6.99–6.91 (m, 1 H, CH-Ar), 3.92–3.84 (m, 2 H, Indol-CH_2_-CH_2_-OH), 2.98 (m, 2 H, Indol-CH_2_-CH_2_-OH), 0.12 (s, 9 H, Si-C(CH_3_)_3_). ^13^C-NMR (100 MHz, Chloroform-d), δ: 158.88, 156.55, 132.92, 128.04, 124.36, 113.42, 111.94, 111.75, 103.97, 63.16, 28.86, 2.06, 0.38.

### Synthesis of (1 s,4 s)-6′-fluoro-N,N-dimethyl-4-phenyl-4′,9′-dihydro-3′H-spiro[cyclohexane-1,1′-pyrano[3,4-b]indol]-4-amine. (Cebranopadol)

#### Method A

The ketone (**7**) (125 mg, 0.66 mmol), was solved in 15 mL of toluene with a catalytic amount of p-toluensulfonic acid; to the solution were added compound (**6**) (149 mg, 0.6 mmol)) and Zeolite K-10 (300 mg). The solution was heated under reflux with a Dean-Stark apparatus for 4 hours. The solvent was removed under vacuum and NaOH 2 N (20 mL) was added to the reaction mixture. The residue was filtered over a celite pad and disolved in AcOEt (20 mL). The organic layers were dried, filtered and concentrated to give a crude product that was purified by flash chromatography (AcOEt/Petroleum ether 2:1) with a yield of 50% as a yellow solid that crystallized in MeOH.

#### Method B

In a two neck round bottom flask, under an argon atmosphere, compound (**6**) (84 mg, 0.336 mmol) was disolved in DCM (10 mL). Compound (**7**) (61 mg, 0.28 mmol), and trimethylsyliltriflate (65 mL, 0.28 mmol) were added whilst stirring at minus 78 °C for 20 hours. The reaction mixture was treated with NaOH 1 N whilst stirring for 30 minutes and was then washed with water (2 X 20 mL). The organic layers were dried, filtered and concentrated under vacuum to obtain a yellow solid that was crystallized in methanol to yield Cebranopadol as a diastereomeric mixture with 90% yield. MS (ESI): [M + H]^+^ = 379.21; HRMS (ESI): [M + H]^+^ Calc. = 379.218018; [M + H]^+^ Found = 379.21809. [M-N(CH_3_)_2_]^+^. = 334.16032; m.p. = 220 °C with decomposition. ^1^H-NMR (400 MHz, Chloroform-d),δ: 8.54 (s, 1 H, NH), 7.37 (m, 5 H, CH-Ar), 7.28 (m, 1 H, CH15), 7.13 (dd, J = 9.6, 2.5 Hz, 1 H, CH18), 6.89 (ddd, J = 9.4, 8.8, 2.5 Hz, 1 H, CH16), 3.96 (t, J = 5.4 Hz, 2 H, -CH_2_ 10), 2.75 (t, J = 5.4 Hz, 2 H, -CH_2_ 9), 2.53 (d, J = 13.7 Hz, 2 H, CH_2_, C6e, C4e), 2.21 (d, J = 12.8 Hz, 2 H, CH_2_, C1a, C3a), 2.10 (s, 6 H, N(CH_3_)_2_), 2.07 (m, 2 H, -CH_2_, CH4a, CH6a), 1.93 (m, 2 H, -CH_2_, CH1e, CH3e). 13C-NMR(100 MHz, Chloroform-d), δ: 159.09 (C-17-Ar), 156.76 (Cq-13), 141.34 (Cq-N-7), 139.06 (Cq, 21), 132.24 (Cq-N-13), 127.57, 127.05, 126.82 (CH-Ar), 125.57 (Cq-indol, 14), 111,57 (CH, 15), 109.74 (CH, 16), 107.31 (Cq, 8), 103.44 (CH, 18), 72.20 (Cq Spiro, 2), 59.81 (CH_2_, 10), 58.79 (Cq, 5), 38.34 (N-CH_3_, 22, 23), 30.93 (CH_2_, 1, 3), 28.35 (CH_2_, 4,6), 22.67 (-CH_2_, 9). ^19^F-NMR: δ: -125.60.

## Electronic supplementary material

A diastereoselective synthesis of Cebranopadol, a novel analgesic showing NOP/mu mixed agonism.
