# Supplementary material for: A diastereoselective synthesis of Cebranopadol, a novel analgesic showing NOP/mu mixed agonism
Source: Sci Rep. 2017 May 25;7:2416. doi: 10.1038/s41598-017-02502-9 (PMC5445067; doi:10.1038/s41598-017-02502-9)

**A diastereoselective synthesis of Cebranopadol, a novel analgesic showing NOP/mu mixed agonism.**

Anna Fantinati*^§^*, Sara Bianco*^§^*, Remo Guerrini*^§^*, Severo Salvadori*^§^*, Salvatore Pacifico*^§^*, Maria Camilla Cerlesi*^#^*, Girolamo Calo’*^#^* and Claudio Trapella* *^§^*

Corresponding Author :

Dr. Claudio Trapella

Department of Chemical and Pharmaceutical Sciences, University of Ferrara, Ferrara, Italy.

Via Fossato di Mortara 17, 44121, Ferrara.

Phone : + 39 0532 455924

Fax : + 39 0532 455953

E-mail: trap@unife.it

**Contents**

Page S4 ^1^H-NMR of compound **3**

page S5 ^13^C-NMR, DEPT-NMR and G-Cosy-NMR of compound **3**

page S6 HMQC-NMR and HMBC-NMR of compound **3**

page S7 ^1^H-NMR of compound **5** page S8 ^13^C-NMR, DEPT-NMR and IR of compound **5**

page S9 ^1^H-NMR of compound **4**

page S10 ^13^C-NMR and DEPT-NMR and HRMS of compound 4

page S11 ^1^H-NMR of compound 7

page S12 ^13^C-NMR and DEPT-NMR and G-cosy of compound 7

page S13 HMBC-NMR and HMQC of compound 7

page S14 IR and HRMS of compound 7

page S15 ^1^H-NMR of compound 6

page S16 ^13^C-NMR and DEPT-NMR of compound 6

page S17 ^1^H-NMR of compound Cebranopadol, method A

page S18 ^13^C-NMR and DEPT-NMR, HMQC-NMR of Cebranopadol, method A

page S19 HMBC-NMR of Cebranopadol, method A

page S20 Roesy-NMR of Cebranopadol, method A

page S21 ^19^F-NMR and HRMS of Cebranopadol, method A

page S22 table 1 of Cebranopadol,method A

page S23 ^1^H-NMR of Cebranopadol, method B

page S24 ^13^C-NMR and DEPT of Cebranopadol, method B

page S25 G-cosy and HMQC-NMR of Cebranopadol, method B

page S26 HMBC-NMR and HMQC-NMR

page S27 HPLC-HRMS of Cebranopadol, method B

**General Information.**

All the NMR spectra were elaborated using Mestre Nova 6.0.2 software and FID data are available on request.

Proton nuclear magnetic resonance (^1^H NMR), carbon nuclear magnetic resonance (^13^C NMR) were recorded using VARIAN 400 MHz . All the spectra were recorded using as solvent CDCl_3_ otherwise the solvent was specified. Infrared (IR) spectra were recorded on a Perkin-Elmer FT-IR Spectrum 100 using as cell zirconium-selenium diamond.

Molecular weights were measured with a mass spectrometer electrospray ESI MICROMASS ZMD 2000 and high resolution spectra with an Agilent ESI-Q-TOF LC/MS 6520 System.

**8-phenyl-1,4-dioxaspiro[4.5]decan-8-ol** **(3).**

^1^H-NMR


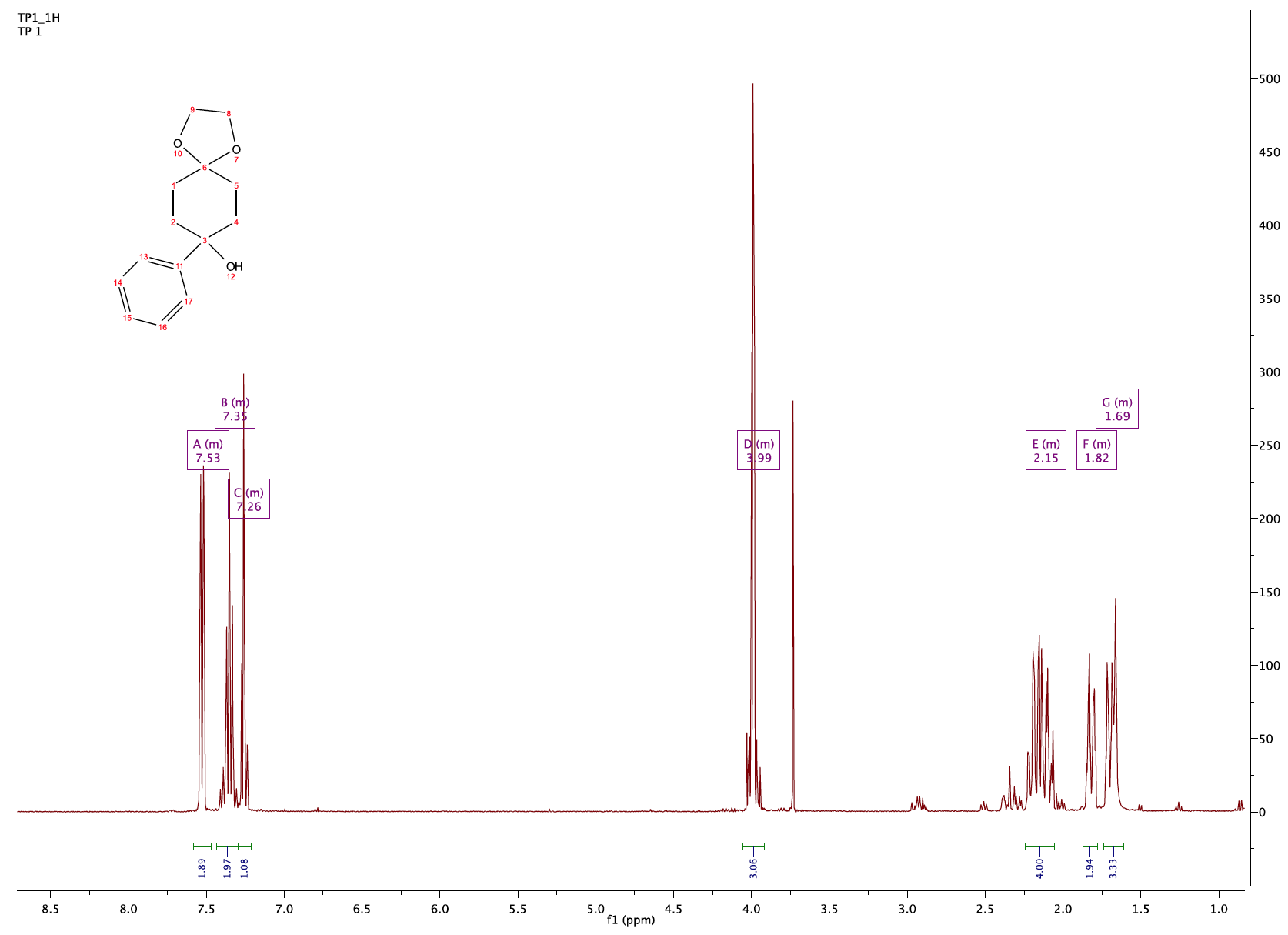


^13^C-NMR and DEPT


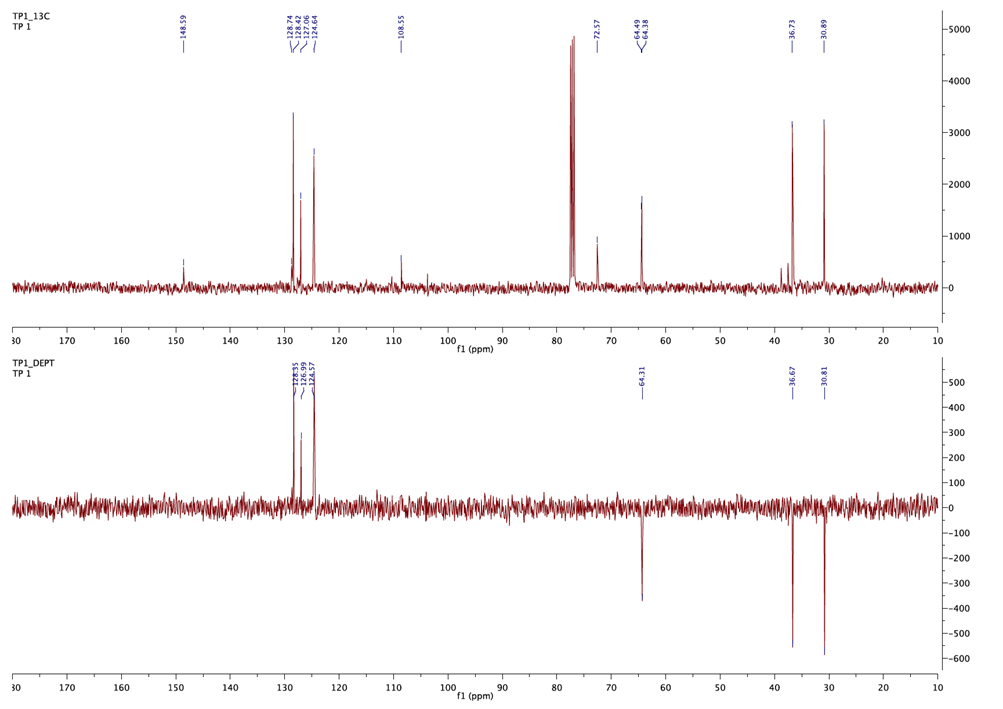


G-Cosy NMR

HMQC

HMBC

**8-azido-8-phenyl-1,4-dioxaspiro[4.5]decane (5).**

^1^H-NMR


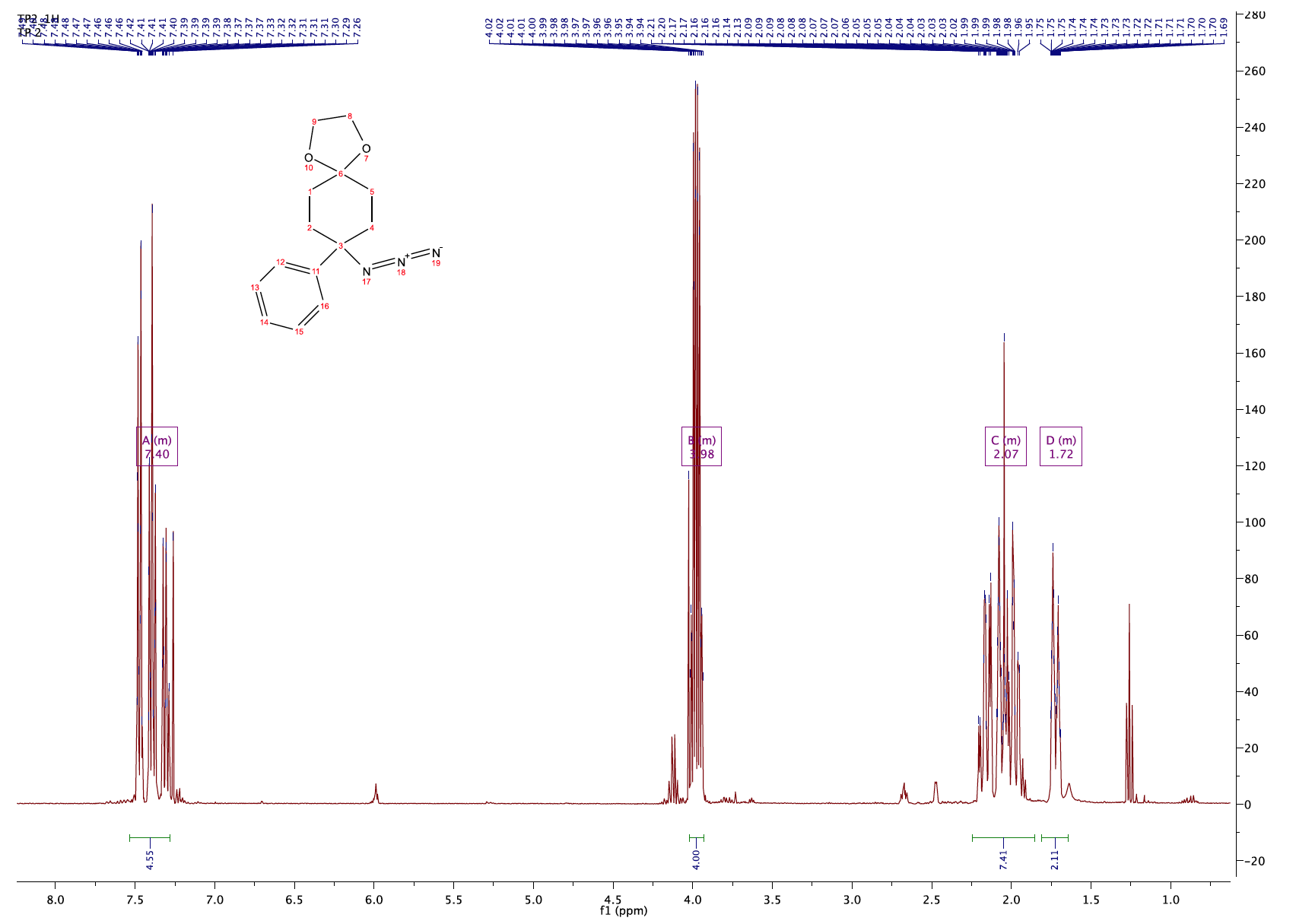


^13^C-NMR and DEPT


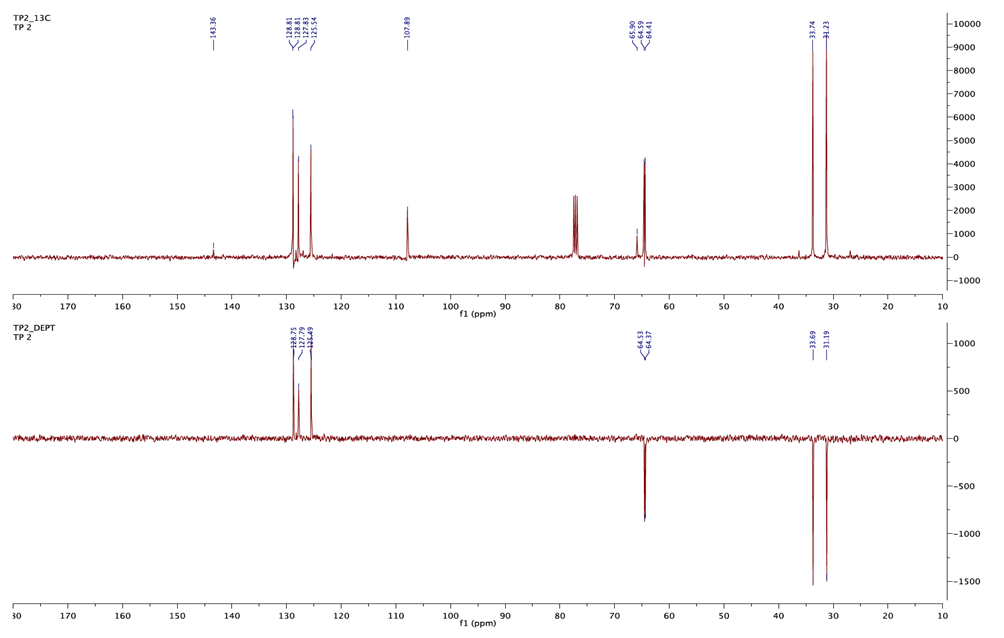


IR

**
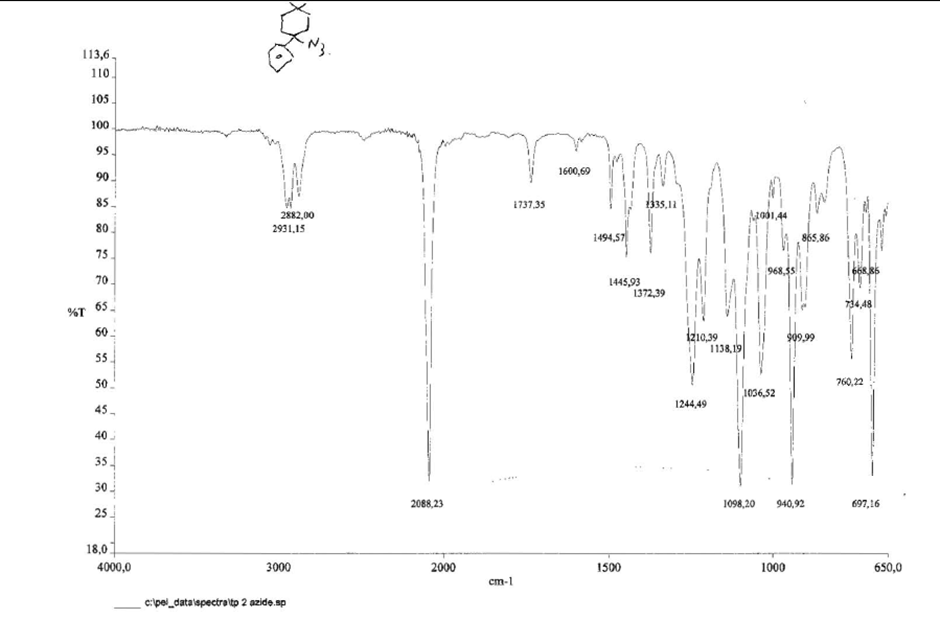
**

**N,N-dimethyl-8-phenyl-1,4-dioxaspiro[4.5]decan-8-amine (4).**

^1^H-NMR

^13^C NMR

HMRS


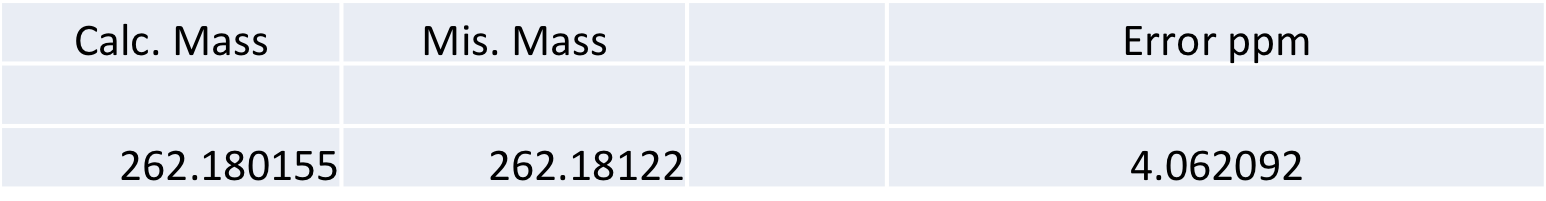


**4-(dimethylamino)-4-phenylcyclohexan-1-one (7).**

^1^H-NMR


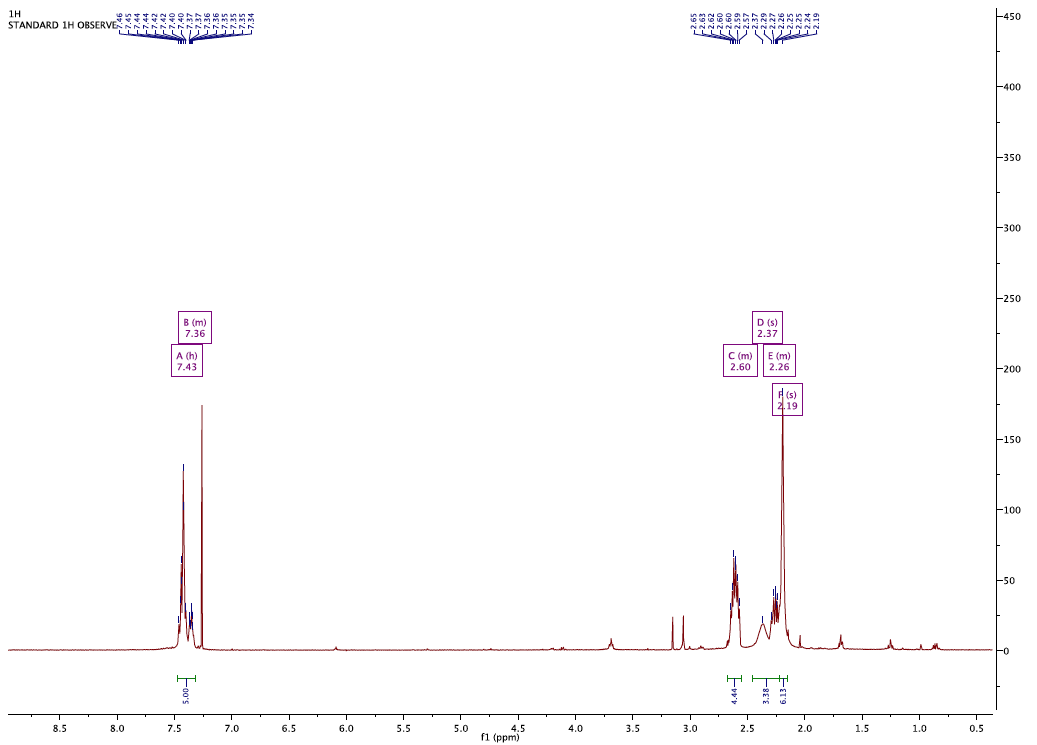


^13^C-NMR


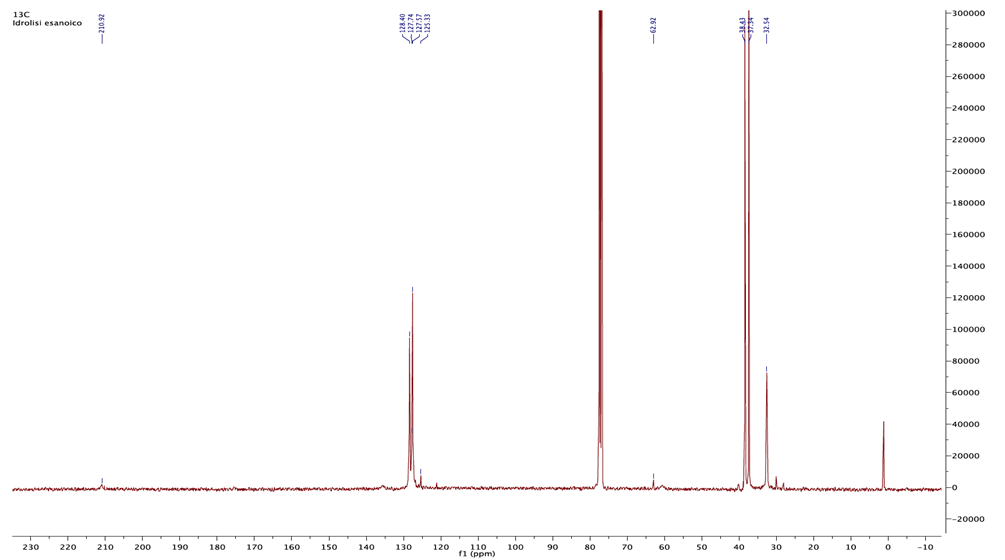


G-COSY
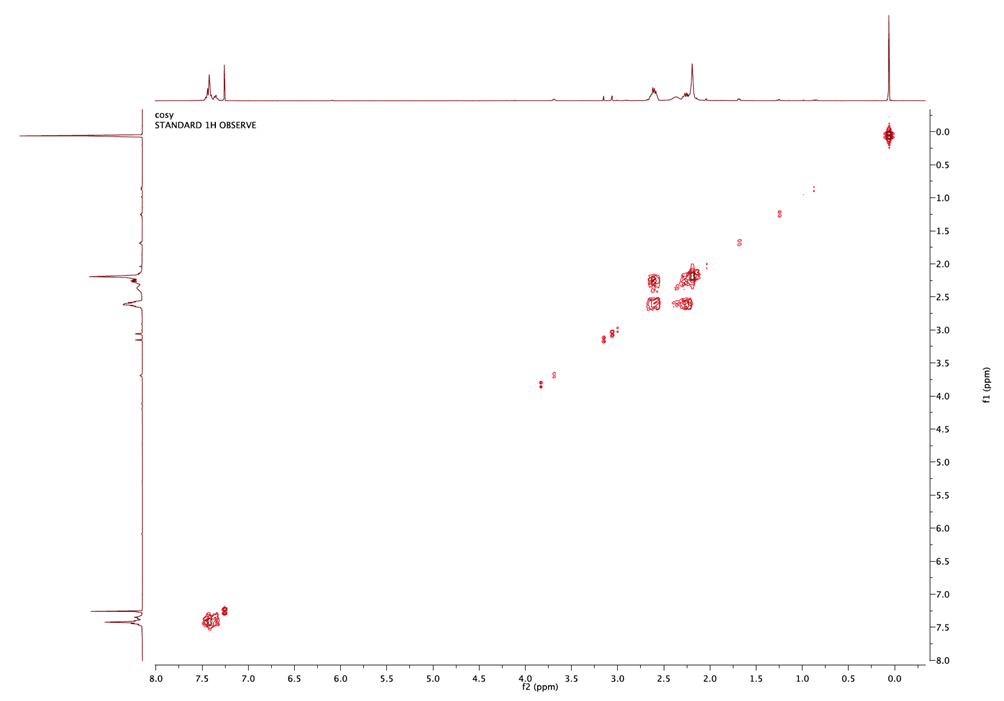


HMBC
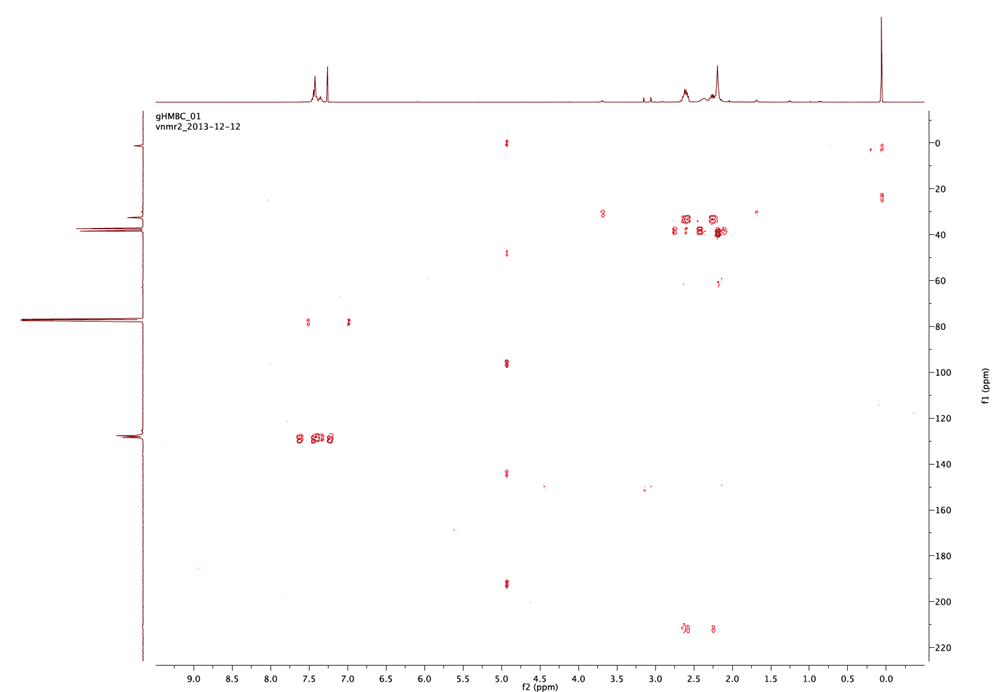


HMQC


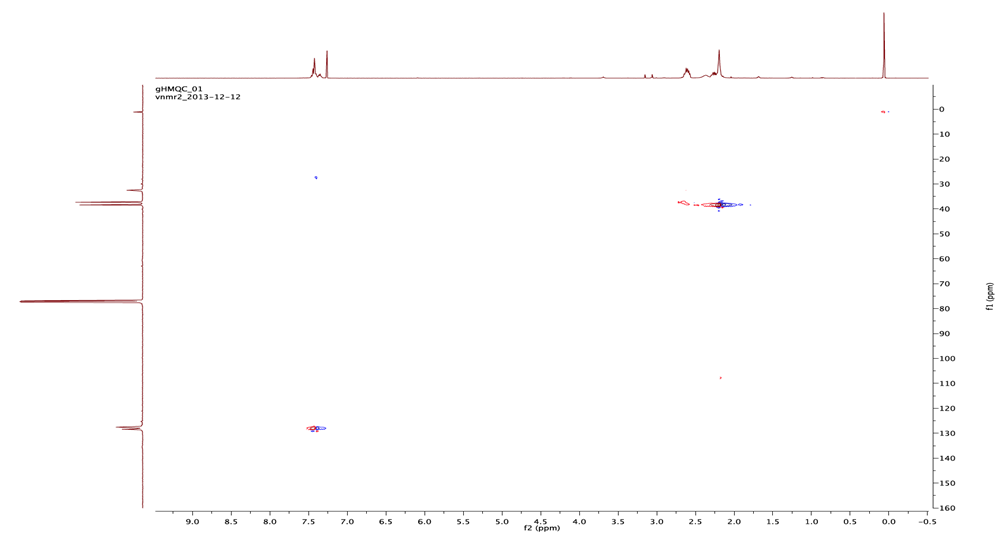


IR

**
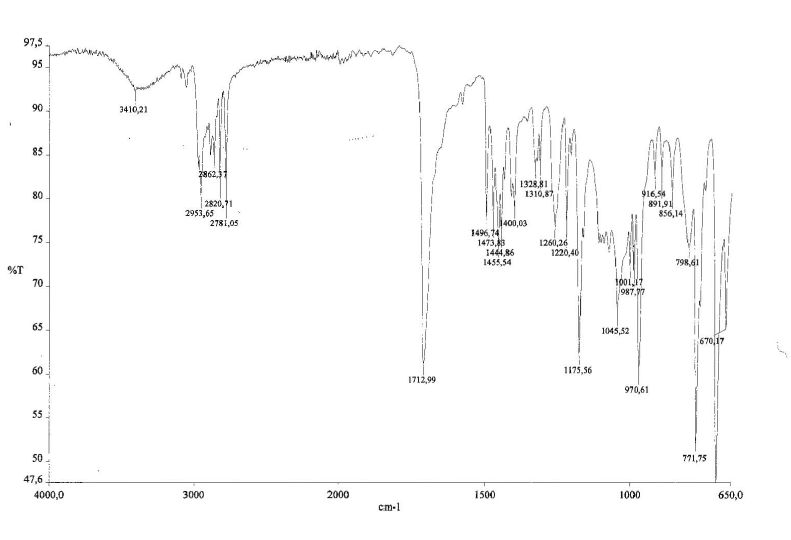
**HRMS

**
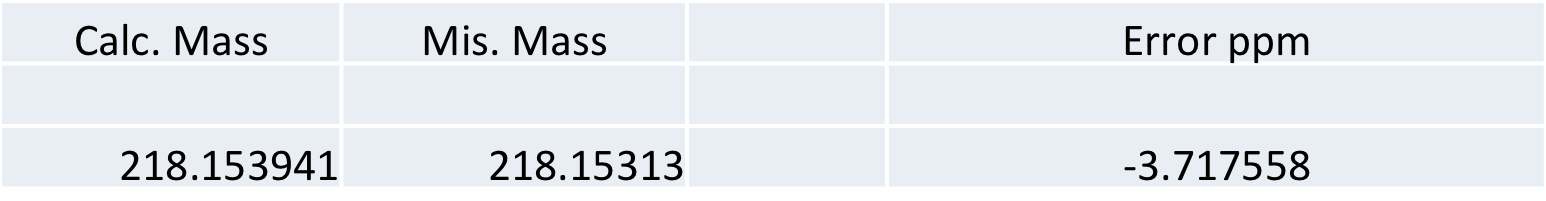
**

**2-(5-fluoro-2-(trimethylsilyl)-1H-indol-3-yl)ethan-1-ol (6).**

^1^H-NMR


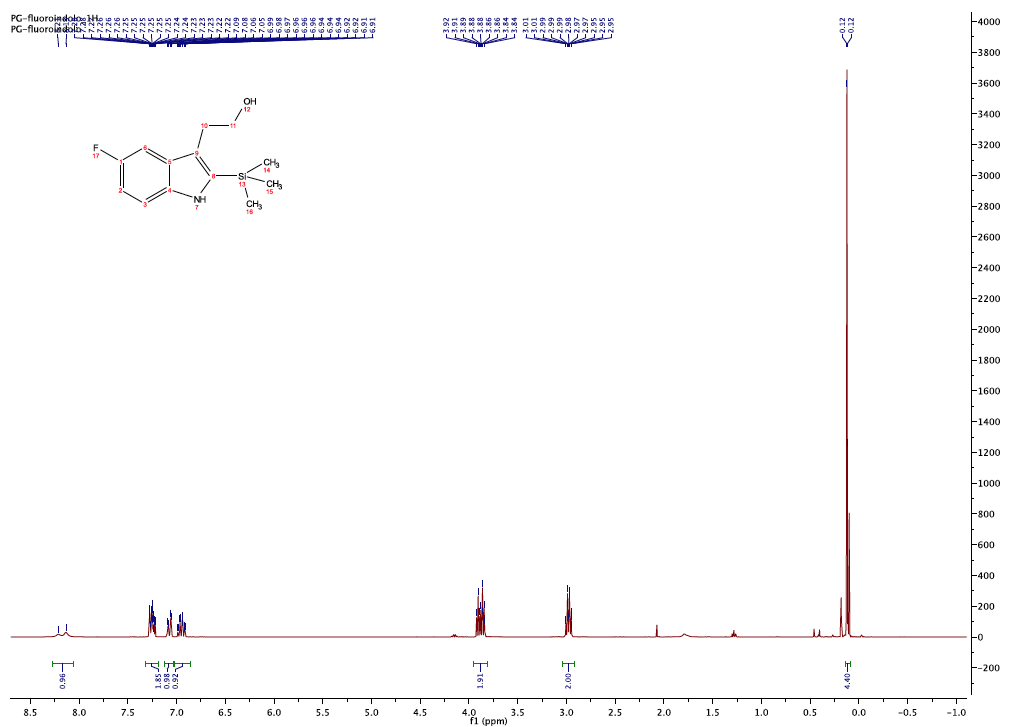


^13^C-NMR and DEPT


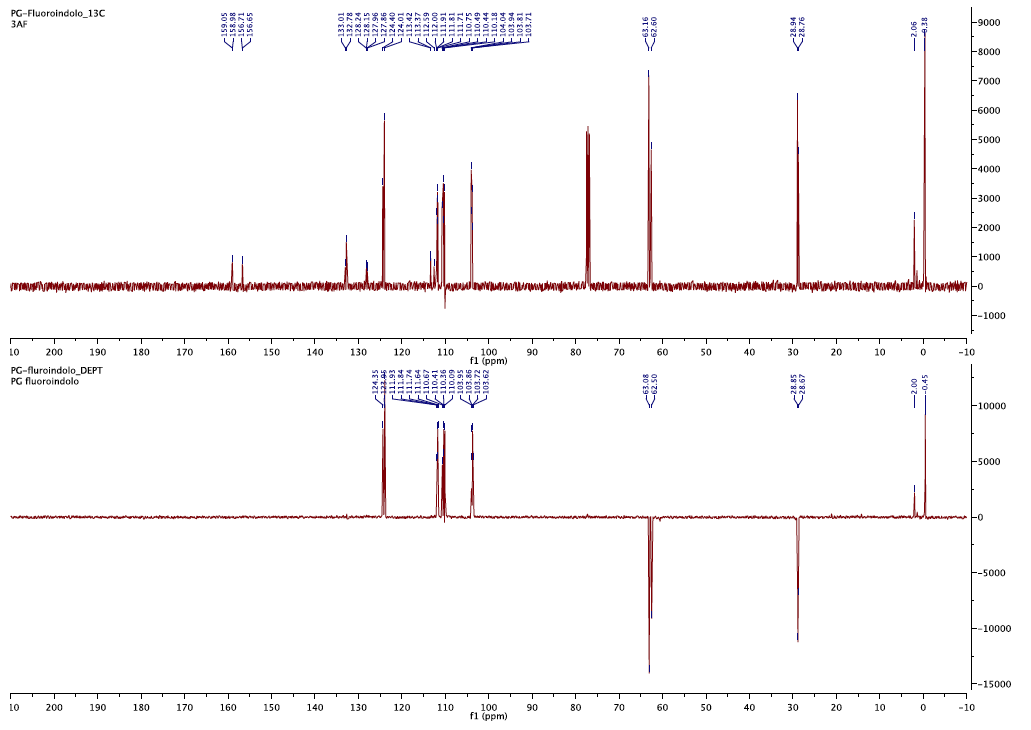


**(1s,4s)-6’-fluoro-N,N-dimethyl-4-phenyl-4’,9’-dihydro-3’H-spiro[cyclohexane-1,1’-pyrano[3,4-b]indol]-4-amine. (Cebranopadol, Method A)**

^1^H-NMR


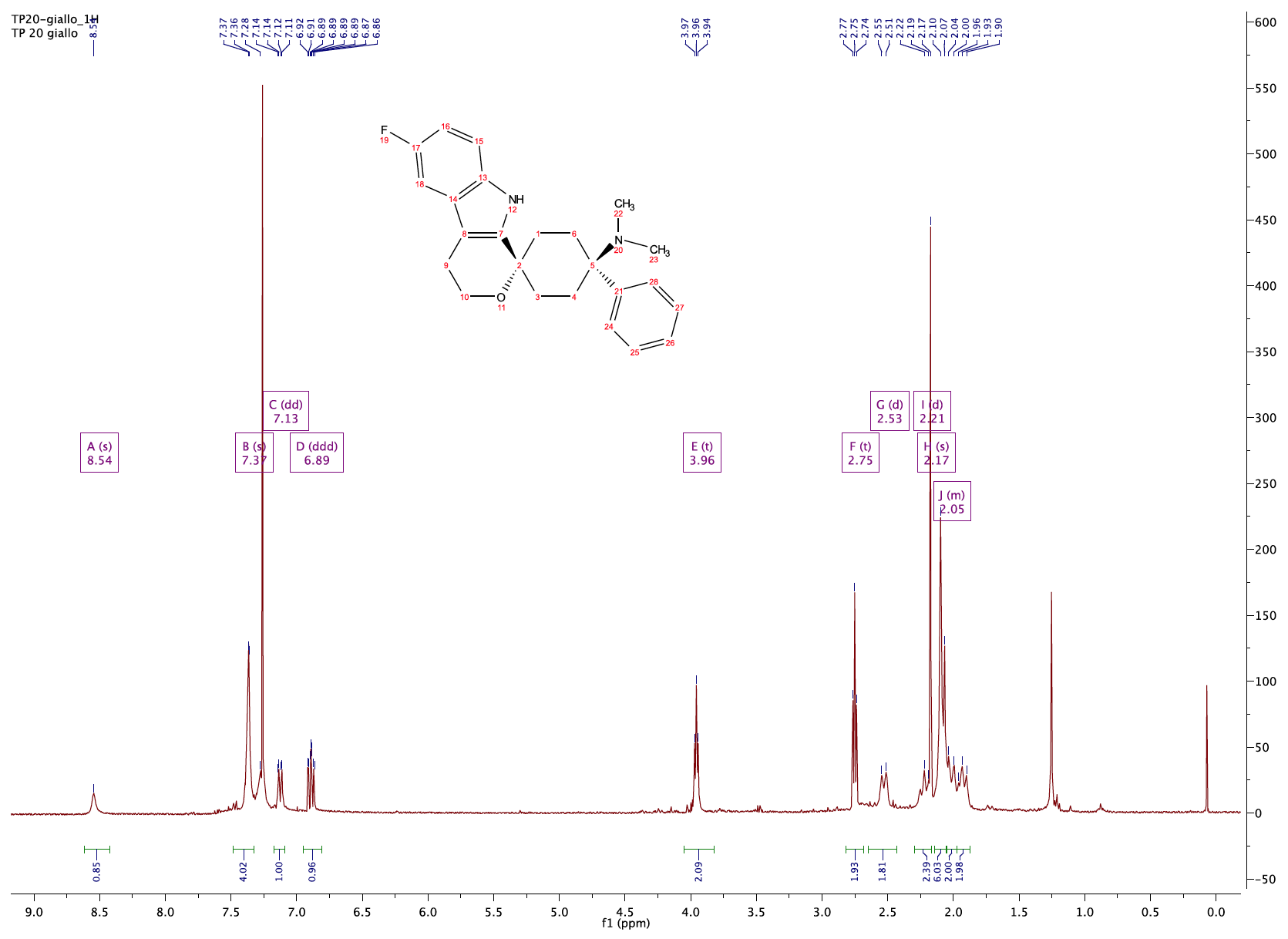


^13^C-NMR and DEPT


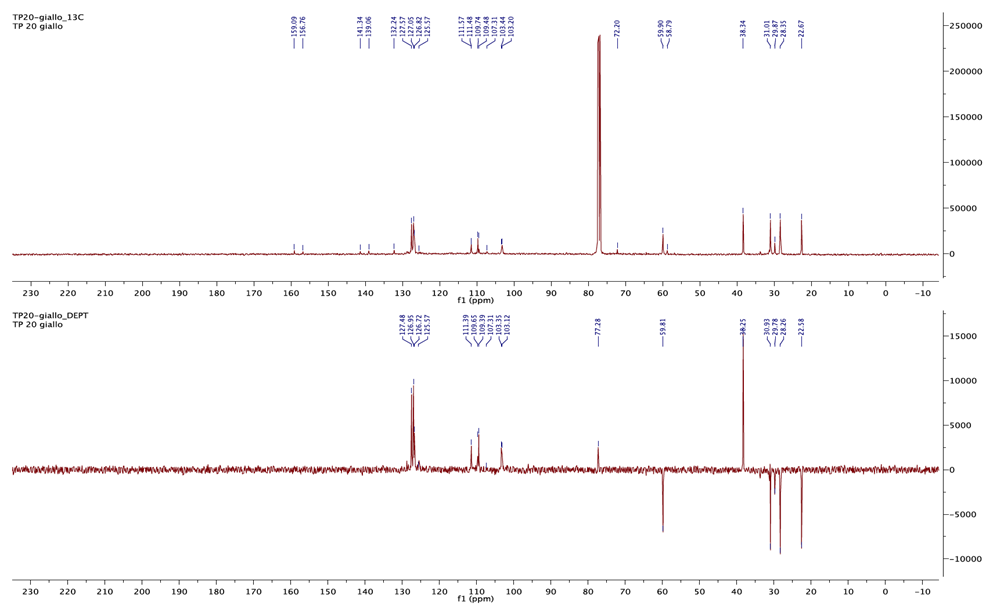


HMQC
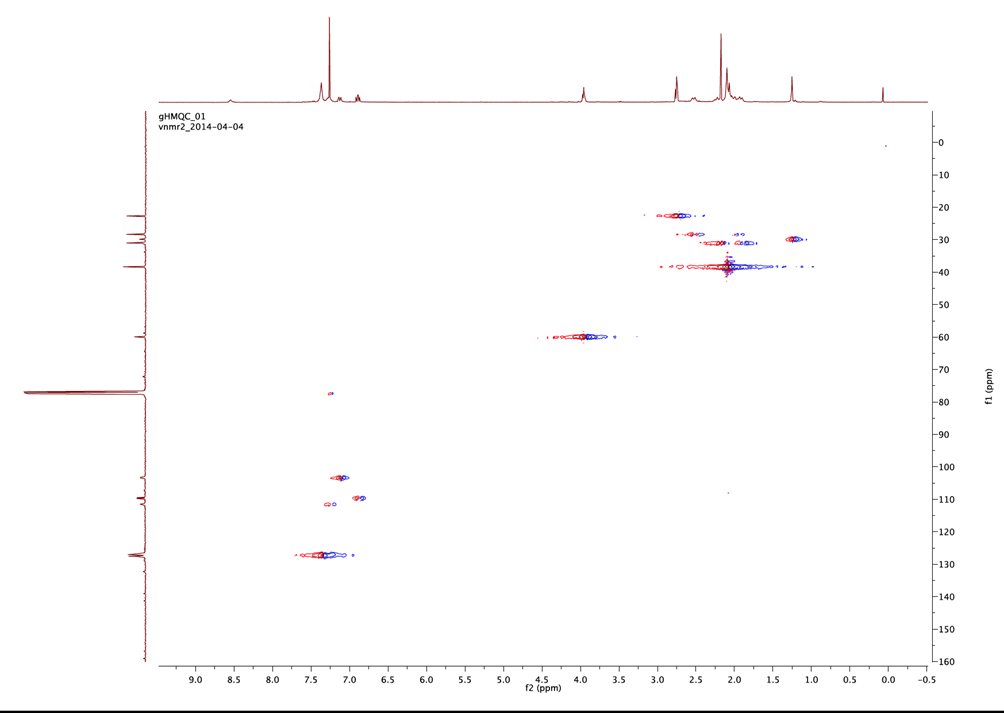


HMBC


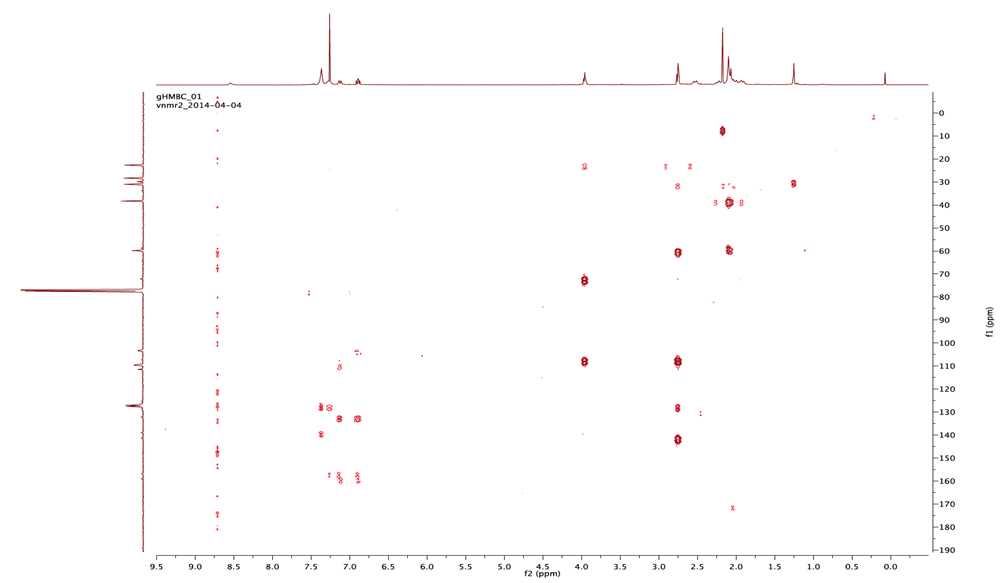


Figure 1

ROESY


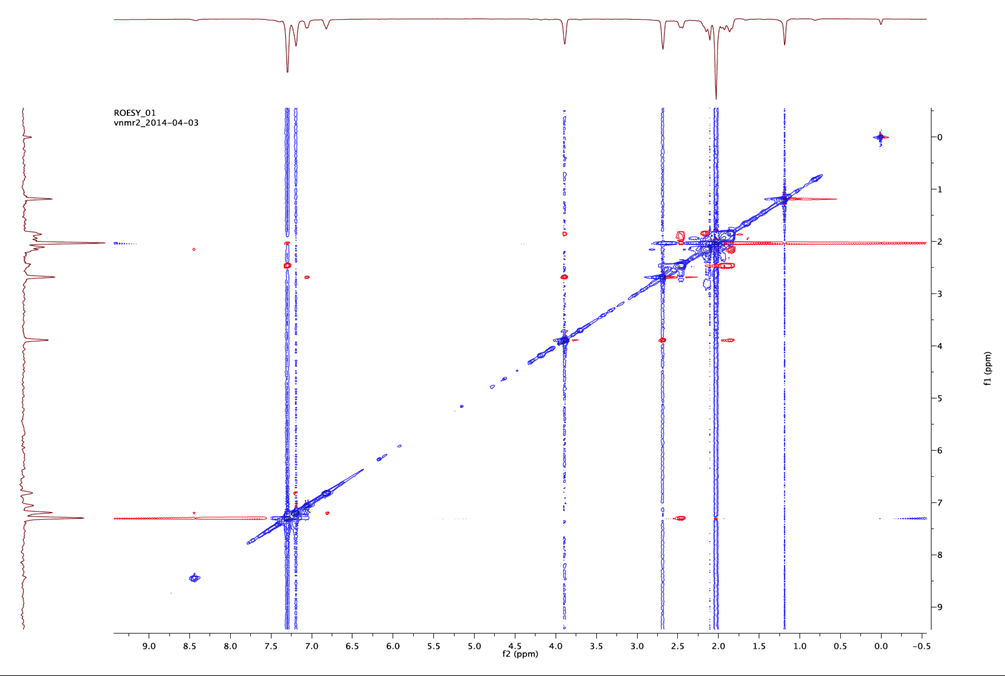


^19^F-NMR


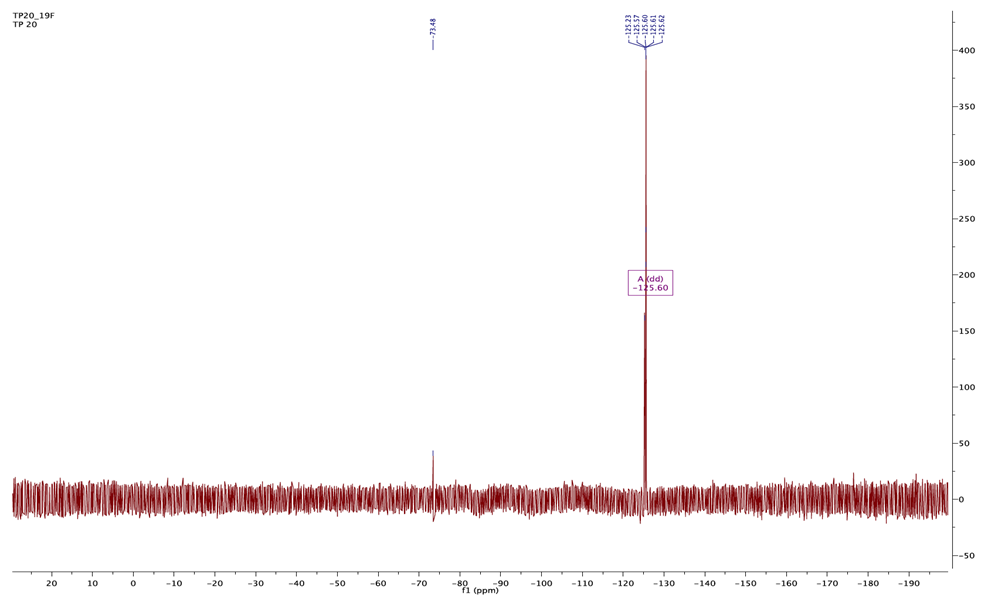


HRMS


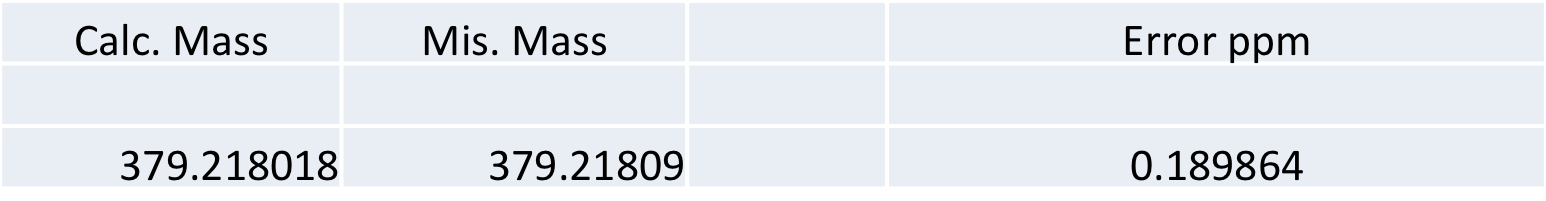


Table 1. Basic pharmacological profile of Cebranopadol in cells coexpressing human recombinant NOP or classical opioid receptors and chimeric G proteins.

|  | **NOP** | | **mu** | | **kappa** | | **delta** | |
| --- | --- | --- | --- | --- | --- | --- | --- | --- |
|  | pEC_50_ | α | pEC_50_ | α | pEC_50_ | α | pEC_50_ | α |
| N/OFQ | 9.59 | 1.00 | inactive | | inactive | | inactive | |
| Fentanyl | inactive | | 8.13 | 1.00 | inactive | | inactive | |
| Dynorphin A | inactive | | 6.67 | 0.82 | 8.54 | 1.00 | 7.73 | 0.99 |
| DPDPE | inactive | | inactive | | inactive | | 8.15 | 1.00 |
| Cebranopadol | 7.28 | 0.89 | 7.20 | 0.99 | 5.98 | 0.55 | 6.31 | 0.81 |

pEC_50_, - log of the concentration of agonist producing half maximal effect. α, maximal agonist effect expressed as fraction of that elicited by the standard agonist i.e. N/OFQ, fentanyl, dynorphin A, and DPDPE for NOP, mu, kappa, and delta receptors, respectively. The data are the mean of at least 4 experiments performed in duplicate.

**(1s,4s)-6’-fluoro-N,N-dimethyl-4-phenyl-4’,9’-dihydro-3’H-spiro[cyclohexane-1,1’-pyrano[3,4-b]indol]-4-amine. (Cebranopadol, Method B)**

^1^H-NMR


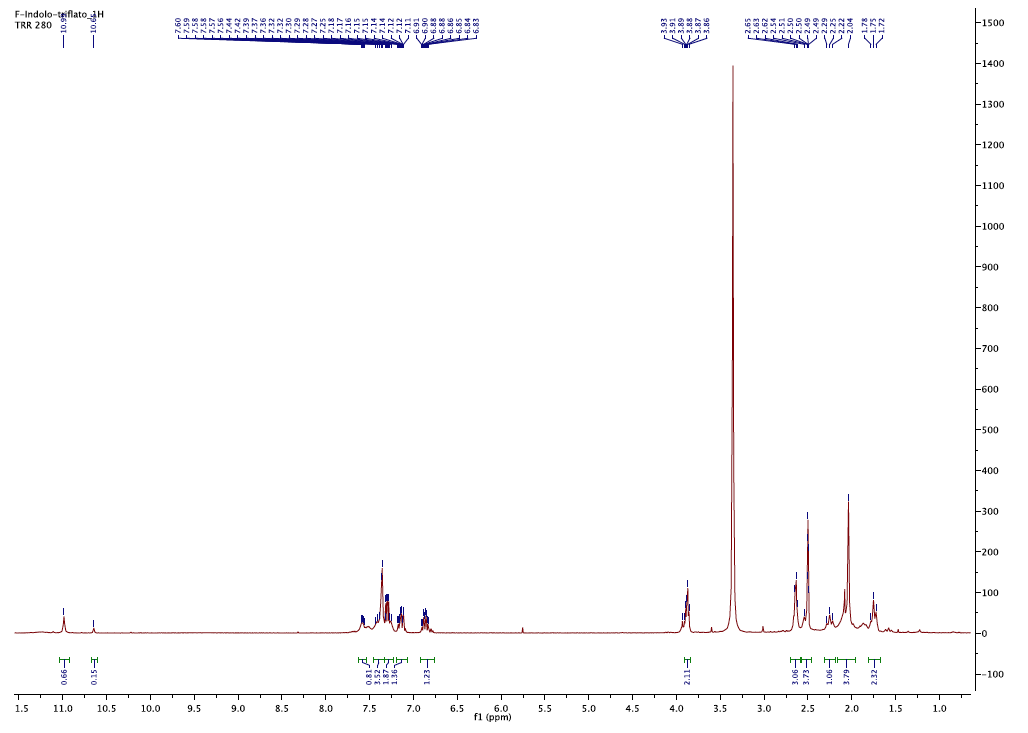


^13^C-NMR and DEPT


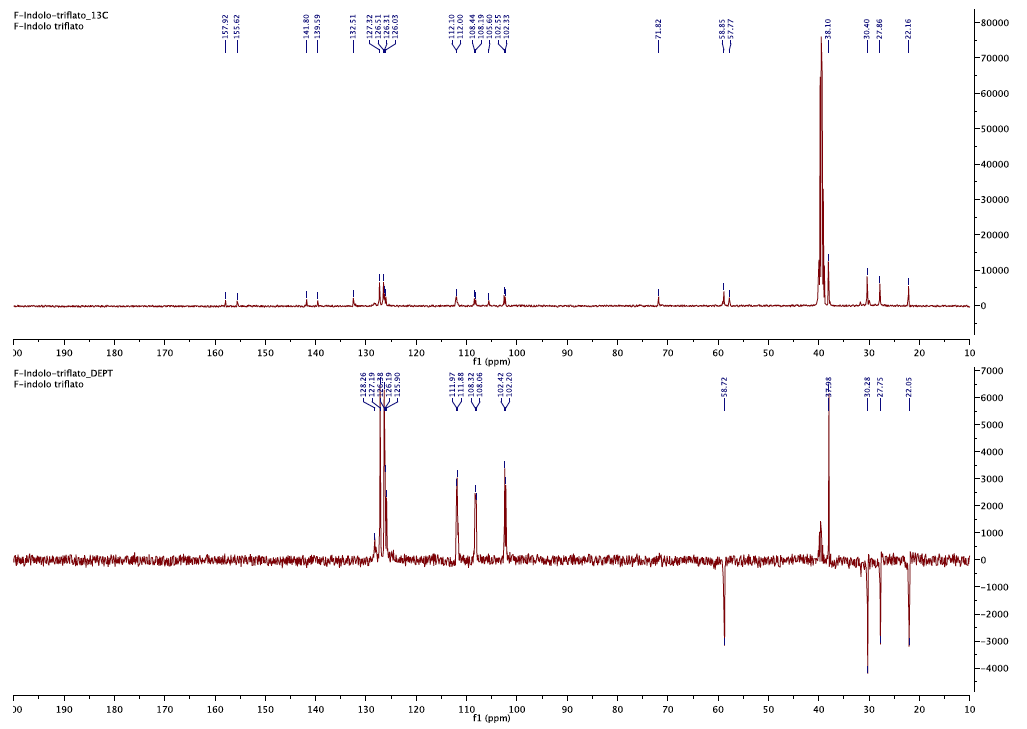


COSY


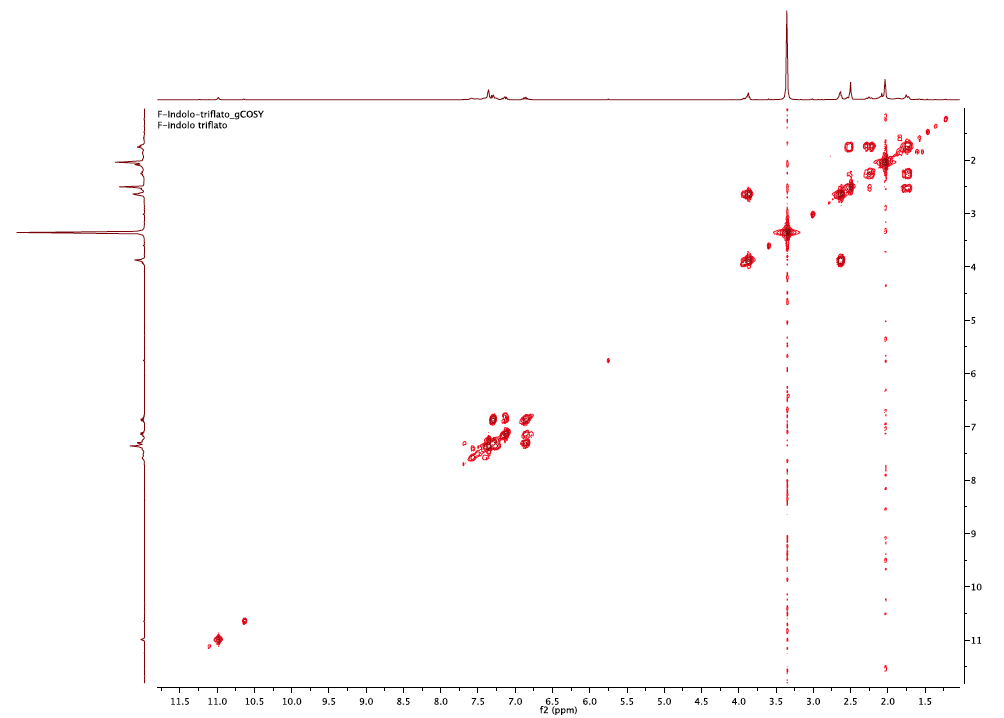


HMQC
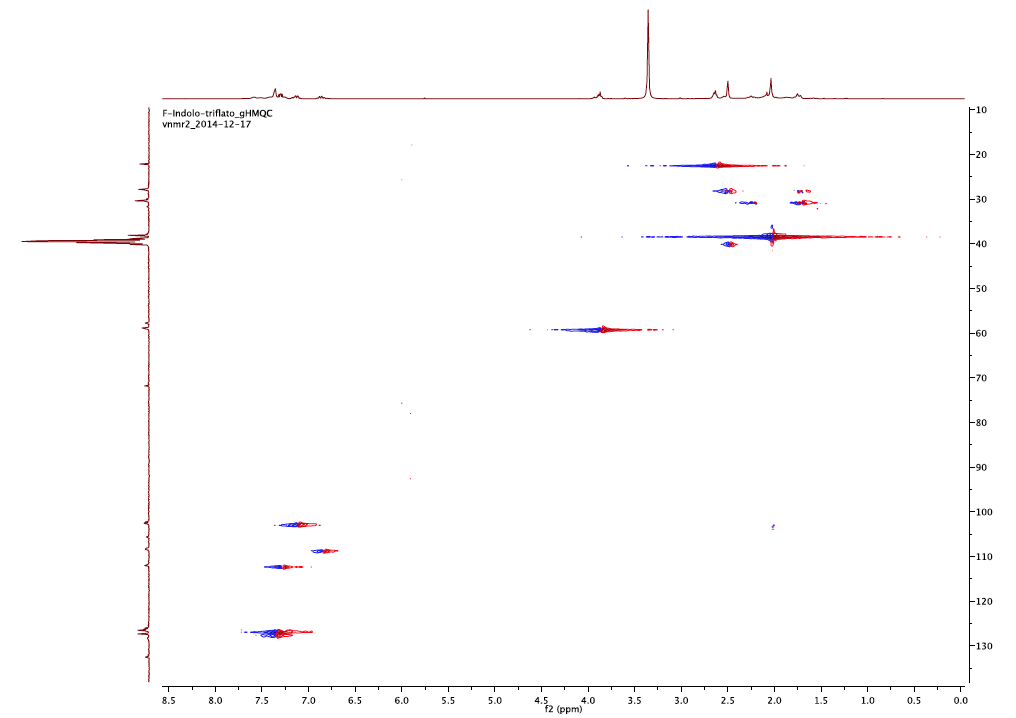


HMBC
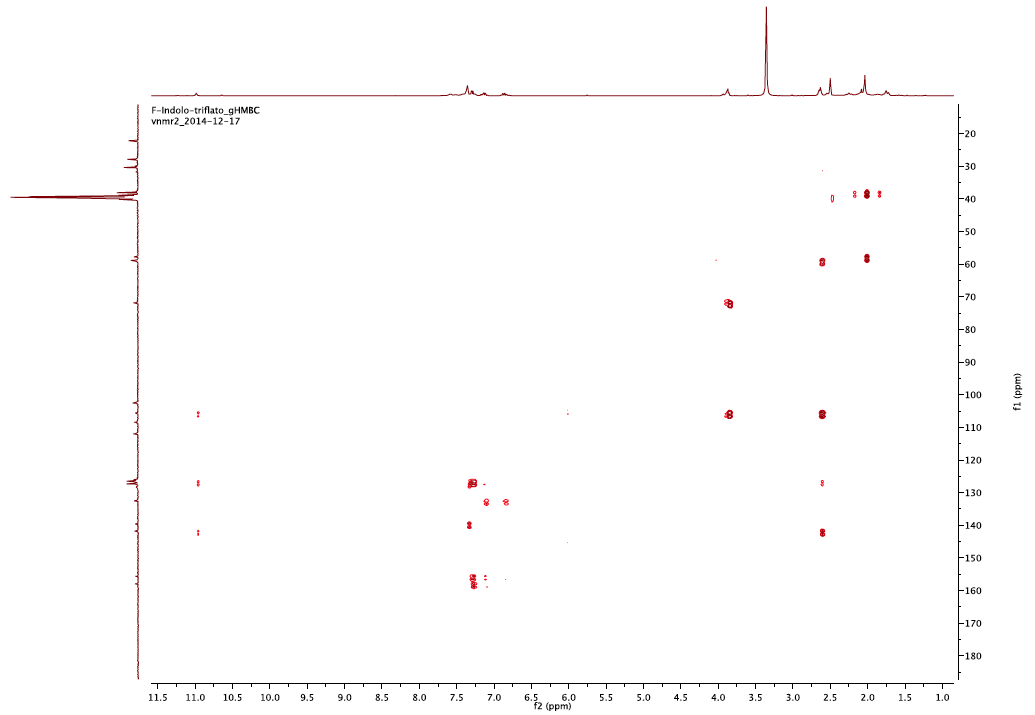


HPLC-HRMS

1H-NMR comparison between crude and pure Cebranopadol


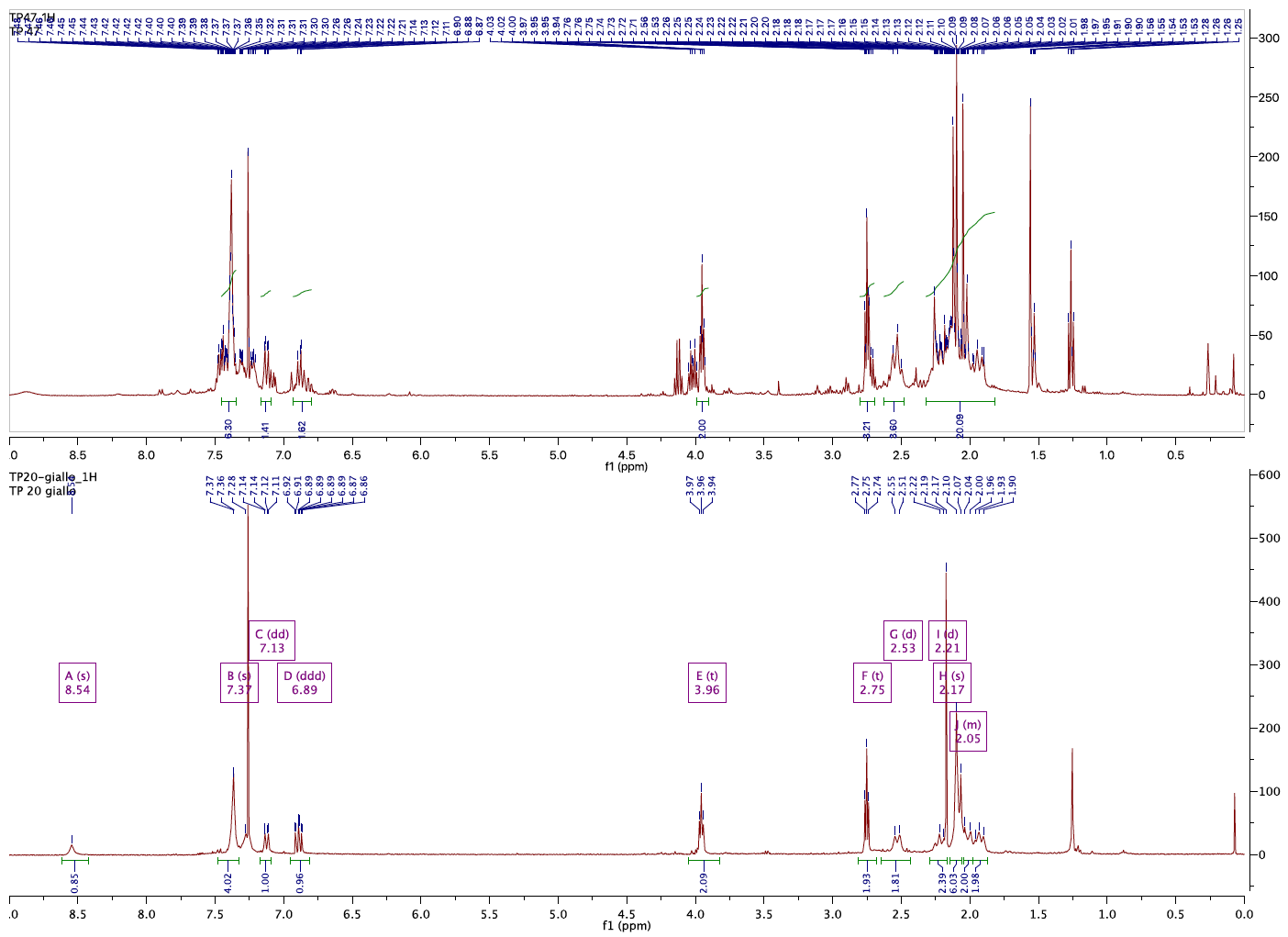


13C-NMR of crude Cebranopadol


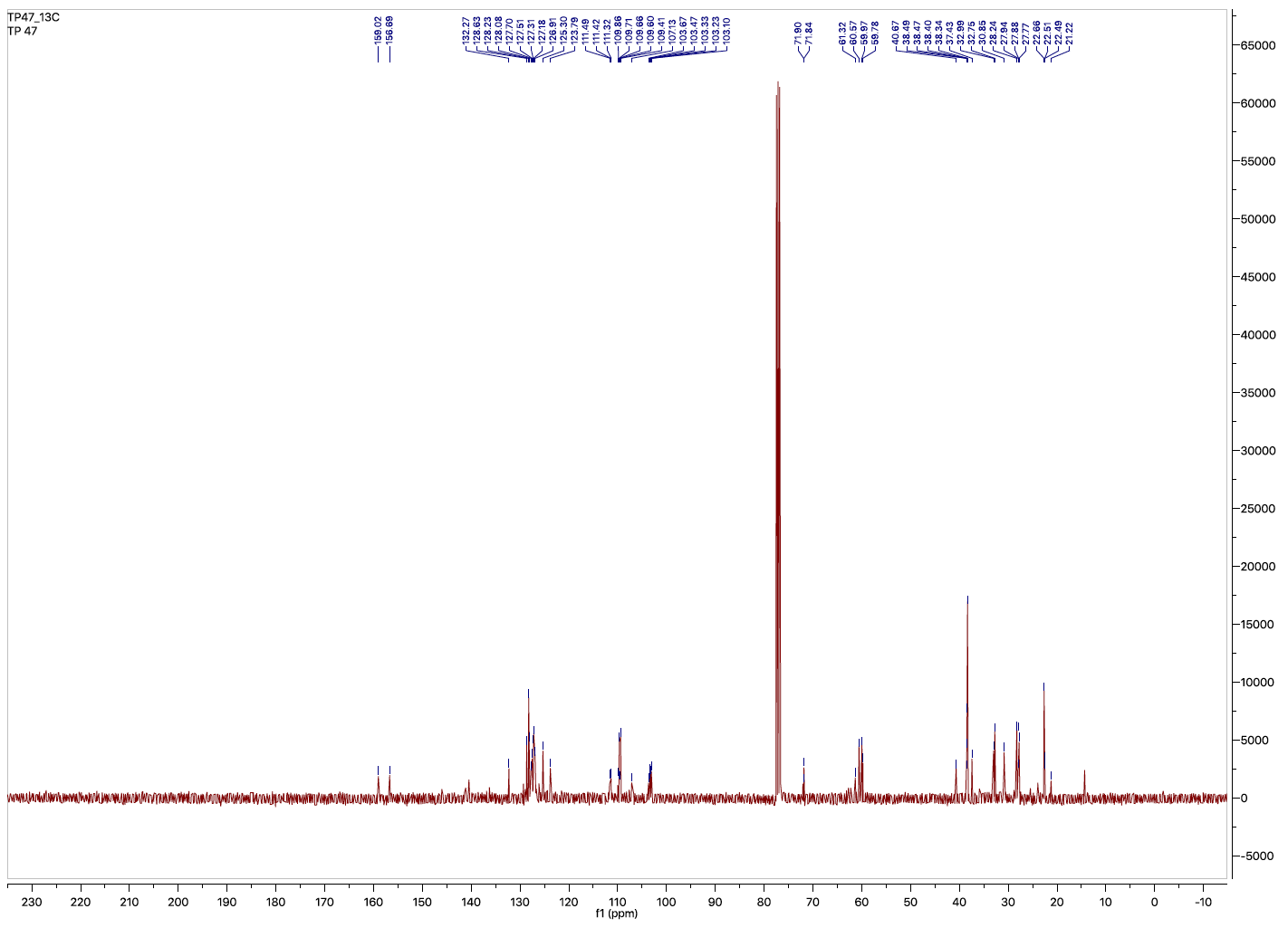


DEPT-NMR Cebranopadol


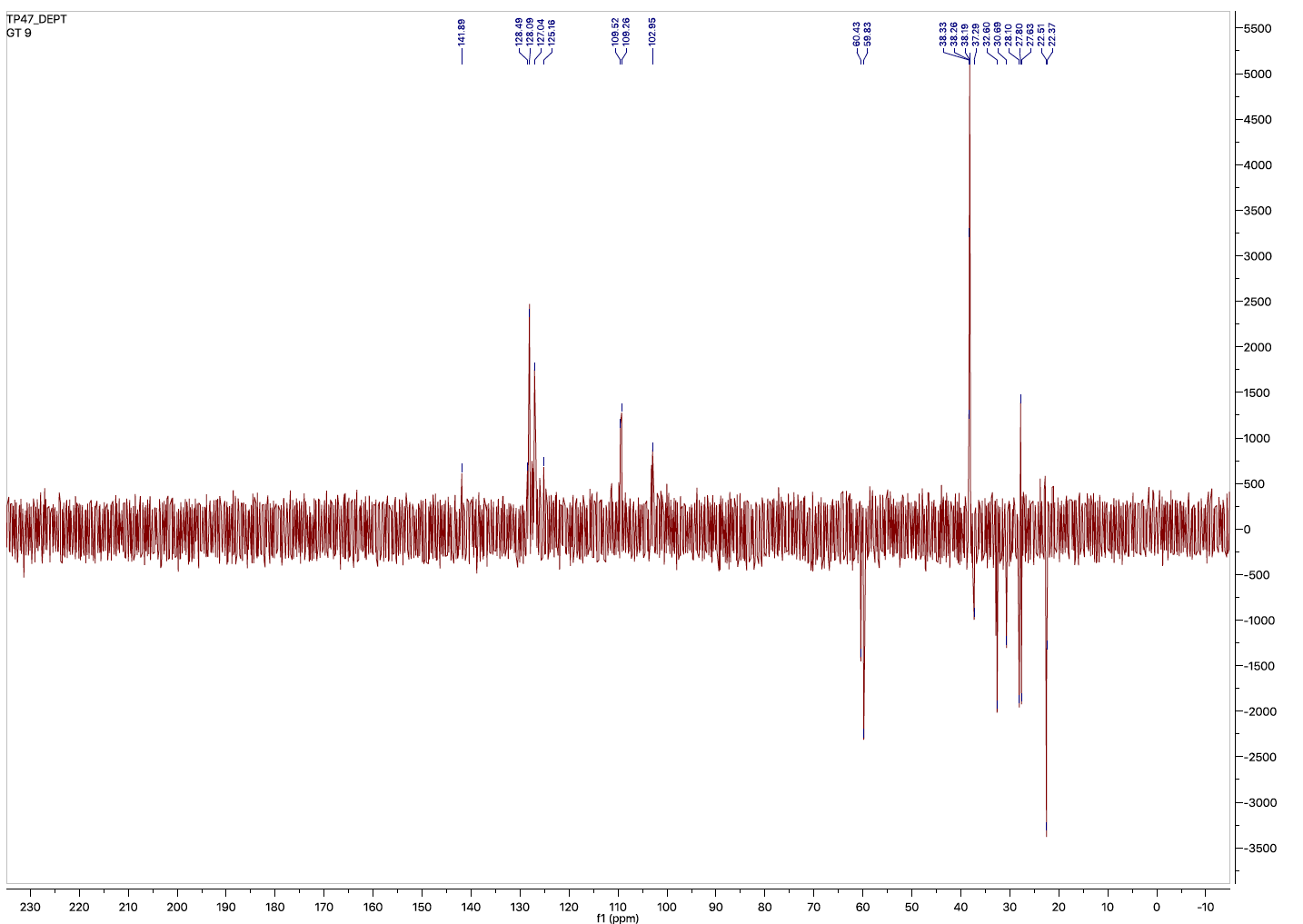


LC-MS spectra of crude Cebranopadol


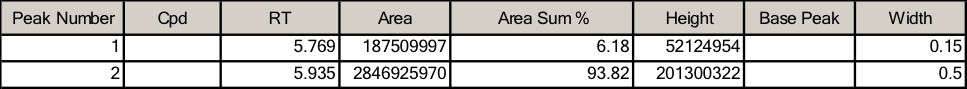

Supplement: Supplementary file 1 — A diastereoselective synthesis of Cebranopadol, a novel analgesic showing NOP/mu mixed agonism. [file 41598_2017_2502_MOESM1_ESM.doc]
